# Water deficit before veraison is crucial in regulating berry VOCs concentration in Sangiovese grapevines

**DOI:** 10.3389/fpls.2023.1117572

**Published:** 2023-02-20

**Authors:** Giacomo Palai, Giovanni Caruso, Riccardo Gucci, Claudio D’Onofrio

**Affiliations:** Department of Agriculture Food and Environment, University of Pisa, Pisa, Italy

**Keywords:** berry aroma, carotenoids, irrigation, terpenes, transcription factors, trascriptome, *Vitis vinifera*

## Abstract

The impact of water deficit on volatile organic compounds (VOCs) responsible for grape aroma remains quite unclear. The aim of this study was to evaluate the effect of different timing and intensity of water deficit on berry VOCs and on their biosynthetic pathways. Fully irrigated control vines were compared with the following treatments: i) two different levels of water deficit from berry pea-size through veraison, ii) one level of water deficit during the lag-phase, and iii) two different levels of water deficit from veraison through harvest. At harvest, total VOC concentrations were higher in berries of water stressed vines from berry pea size through veraison or during the lag phase, whereas post-veraison water deficit determined similar concentrations as control. This pattern was even more pronounced for the glycosylated fraction and was also observed for single compounds, mainly monoterpenes and C_13_-norisoprenoids. On the other hand, free VOCs were higher in berries from lag phase or post-veraison stressed vines. The significant glycosylated and free VOCs increment measured after the short water stress limited to the lag phase highlight the pivotal role played by this stage in berry aroma compound biosynthesis modulation. The severity of water stress before veraison was also important, since glycosylated VOCs showed a positive correlation with the pre-veraison daily water stress integral. The RNA-seq analysis showed a wide regulation induced by irrigation regimes on terpenes and carotenoids biosynthetic pathways. The terpene synthases and glycosyltransferases as well as genes of the network of transcription factors were upregulated, especially in berries from pre-veraison stressed vines. Since the timing and intensity of water deficit contribute to regulate berry VOCs, irrigation management can be used to achieve high-quality grapes while saving water.

## 1 Introduction

As hot and dry summers are occurring more and more frequently, the importance of irrigation in many wine-grape regions is rising. The concomitantly long drought periods and the competition for water resources require the adoption of sustainable irrigation strategies to reconcile the mitigation of stress with high water use efficiency. In addition, water deficit is often developing early, which makes irrigation crucial during most of the vine vegetative and reproductive cycle to preserve grape yield and quality.

Many studies have been conducted so far to elucidate how vine water status affects secondary metabolites that confer important quality features to grapes and wines (Van Leeuwen et al., 2009; Casassa et al., 2015; Gambetta et al., 2020; Romero et al., 2022). Changes in the regulation of the biosynthesis of phenolic compounds, such as anthocyanins, flavonols, stilbenes, and tannins, induced by deficit irrigation have been widely investigated in grapevines (Deluc et al., 2009; Savoi et al., 2017; Ju et al., 2019; Palai et al., 2022a). Vine water status also affects grape aroma (Escalona and Flexas, 1999; Chapman et al., 2005; Des Gachons et al., 2005; Koundouras et al., 2009), but the impact of water deficit on berry VOCs remains quite unclear. Song et al. (2012) reported higher concentrations of glycosylated C_13_-norisoprenoids (β-damascenone, 3-hydroxy-β-damascenone, 1,1,6-trimethyl-1,2-dihydronaphthalene, and 3-oxo-α-ionol) in Merlot berries from deficit irrigated vines (35%–70% of crop evapotranspiration, ET_c_) with respect to fully irrigated ones. The higher concentration of β-damascenone was also measured in Merlot wines produced from deficit irrigated vines (35% ET_c_) (Qian et al., 2009). Volatile benzene derivatives and phenol compounds, such as benzaldehyde, guaiacol, 4-ethylphenol, and 4-vinylphenol, increased in berries from deficit (35% ET_c_) and full (100% ET_c_) irrigated vines (cv. Bobal) with respect to those from rainfed ones (Lizama et al., 2021), whereas higher concentrations of acetaldehyde and isoamyl acetate were measured in wines obtained from rainfed vines (cv. Godello) than in those from irrigated vines (Mirás-Avalos et al., 2019). Consistent results for volatile phenols and C_6_ compounds were obtained in a similar experiment conducted on cv. Verdejo (Vilanova et al., 2019). Vine water status affected esters in Cabernet Sauvignon berries contributing to desirable fruity and floral notes: supplying 25% or 50% ET_c_ enhanced esters concentration with respect to fully irrigated vines (Torres et al., 2022), whereas they were depressed at stem water potential (Ψ_stem_) lower than −1.0 MPa (Talaverano et al., 2017).

In many varieties, a key role in berry aroma profile is played by terpenes. Some monoterpenes such as linalool, geraniol, and α-terpineol are key compounds in determining the floral sensory attributes of most wine grape varieties (Robinson et al., 2014). The concentration of glycosylated terpenes, such as nerol and geraniol, increased by increasing the level of water stress in Merlot berries (Song et al., 2012; Palai et al., 2022b), whereas the opposite was observed for free α-terpineol and geraniol, in berries of Treixadura (Bouzas-Cid et al., 2018). The timing when water deficit was imposed represented a further factor able to influence the content of terpenes at harvest, potentially clarifying some contradictory evidence reported in the literature. A higher concentration of glycosylated VOCs, especially monoterpenes and linalool derivatives, was measured in Sangiovese berries from vines stressed before veraison, whereas negative or negligible effects were observed when deficit was applied after veraison (Palai et al., 2022b). Similarly, in Tocai Friulano, Savoi et al. (2016) reported that pre-veraison water deficit enhanced the accumulation of free nerol, linalool, hotrienol, and α-terpineol and the expression of terpene synthase 35 and 54 through the methylerythritol phosphate pathway. Transcriptional regulation also enhanced monoterpenes accumulation overexpressing terpene synthases in berries from Viognier vines subjected to both early deficit and sustained deficit irrigation (Wang et al., 2019). The same authors reported that the concentration of free α-terpineol and free linalool was higher only when water deficit developed before veraison, whereas prolonging water stress until ripening did not have the same effect. Furthermore, many of the transcription factors (TFs) characterizing the complex network that regulates the terpenes pathways have been proposed as regulatory candidates for the terpenes biosynthesis under drought and abiotic stress, especially genes of MYB, NAC, and WRKY families (Wong et al., 2016; Costantini et al., 2017; Zhu et al., 2022).

This work aimed at elucidating the effects of water deficit on berry VOCs, assuming that different levels of water stress applied at different stages of berry development are able to regulate the berry aroma compounds biosynthesis. In particular, we tested the hypothesis that water deficit applied before veraison, and especially during the lag phase, plays a key role in regulating biosynthetic pathways of berry aroma compounds. In this experiment, we compared a fully irrigated control against five deficit irrigation treatments consisting of applying different levels (moderate or severe) of water stress at different stages during berry development (from the green phase to veraison, during the lag phase, and after veraison), over two consecutive growing seasons.

## 2 Materials and methods

### 2.1 Plant material and experimental conditions

The experiment was conducted using 7-year-old grapevines (*Vitis vinifera* L. cv. Sangiovese) grafted on 110 Richter (*Vitis berlandieri* × *Vitis rupestris*) at the experimental farm of the Department of Agriculture Food and Environment of the University of Pisa in 2019 and 2020. Vines were grown in rows under open field conditions in 50-L containers (40% peat and 60% silty-loam soil), spaced at 4.2 m × 0.9 m, and trained to a Guyot system as previously reported (Palai et al., 2022a). Fertilizers were supplied in spring by irrigation until the irrigation treatments were started (Palai et al., 2022a). Berry growth was monitored using a modified Eichhorn–Lorenz (E–L) scale (Coombe, 1995). The climatic conditions and phenological stages were reported in Palai et al. (2022a). In brief, annual precipitations (970 and 1,060 mm in 2019 and 2020, respectively) and reference evapotranspiration (900 and 891 mm, respectively) were similar between years, whereas the mean air temperature in April and May was lower in 2019 than 2020 (14.7°C and 16.5°C, respectively, average between months), determining a 7-day delay in fruit set. The harvest date for each irrigation regimes was established according to a total soluble solids (TSS) threshold (22 ± 0.5° Brix) to prevent effects due to soluble carbohydrates concentrations on berry-glycosylated VOCs concentration or on berry dry weight (DW).

### 2.2 Irrigation and vine water status

The irrigation volumes were supplied *via* drip lines in two or three shifts a day and were extensively detailed in Palai et al. (2022a). All vines were fully irrigated until the berries reached pea-size stage (DOY 163 and 156 in 2019 and 2020, respectively), when irrigation was differentiated as follows: I) control vines (FI), which received 100% of water needs (446 and 443 L per vine during the entire irrigation period in 2019 and 2020, respectively); II–III) a severe (S) and a moderate (M) regulated deficit irrigation (RDI) from berry pea size to the beginning of veraison (RDI-1S, 30%–31% of FI, and RDI-1M, 54%–56% of FI, respectively); IV) a severe water deficit applied during the lag phase (RDI-LS, 38% of FI, in both years); and V–VI) a severe and a moderate RDI from the beginning of veraison to harvest (RDI-2S, 31%–38% of FI; RDI-2M, 48%–57% of FI, respectively). All RDI vines were fully irrigated when not subjected to water deficit. Irrigation of control vines was adjusted based on vines’ actual water consumption and to maintain stem water potential (Ψ_stem_) above approximately −0.5 MPa (Caruso et al., 2022). Vine water status was determined through Ψ_stem_ measured on five vines per treatments, at 7–10-day intervals from the irrigation differentiation until harvest (13 times in both years) by a Scholander pressure chamber (PMS Instruments, Albany, OR, USA) following the protocol reported in Palai et al. (2021). The water stress integral (WSI) was then calculated from Ψ_stem_ data, as reported in Myers (1988), and the daily WSI (dWSI) was determined from berry pea size to lag phase (PS-L), from lag-phase to the beginning of veraison (L-V), from the beginning of veraison to harvest (V-H), and from pea-size to harvest (PS-H).

### 2.3 Yield and berry characteristics

Each vine was harvested individually, and clusters were immediately weighed for yield determination. Yield was affected by water availability: the highest (3.161 and 3.232 kg per vine in 2019 and 2020, respectively) and lowest (2.199 and 1.831 kg per vine in 2019 and 2020, respectively) values were recorded in FI and RDI-1S vines, respectively, in both years (Palai et al., 2022a). Berry fresh (FW) and dry weight (DW) were determined on three samples of 100 berries per irrigation treatment, measured within 1 h from harvest and after oven-drying at 70°C until constant weight, respectively. The berry skin to pulp ratio and the specific skin weight (SSW) were determined at veraison, during berry maturation (DOY 224 and 240 in 2019 and DOY 218, 231, and 244 in 2020), and at harvest for all irrigation treatments. The skin was manually separated from the pulp, weighed, and the skin to pulp ratio calculated separately from a total of 90 berries per treatment. The berry skin area was determined by measuring the longitudinal and transversal diameters of each berry with a digital caliper and then calculated by assimilating the berry shape to an ellipsoid. The SSW was determined as grams of skin per cm^2^. Berry TSS, titratable acidity (TA), and pH were determined on three samples per treatment (30 berries for each sample). The berry TSS were measured with a digital refractometer (DBRwine, HM digital Ltd., Seoul, Korea), whereas a 10-ml aliquot of juice was used to measure the pH (Hanna Instruments, Woonsocket, RI, USA) and then titrated with 0.1 N NaOH to an endpoint pH of 8.2 to determine TA (g L^−1^ of tartaric acid).

### 2.4 Berry VOC determination

Three samples of 100 berries each were randomly collected at harvest from each irrigation treatment and used for free and glycosylated VOCs extraction by solid phase extraction (SPE), as previously reported (D’Onofrio et al., 2018). Chromatographic analyses were carried out using an Agilent 7890A gas chromatograph coupled with an Agilent 5975C quadrupole mass spectrometer. The capillary column was an HP-Innowax (30 m length, 0.25 mm i.d., and 0.25 mm film thickness) from Agilent (Waldbron, Germany). Helium was used as carrier gas with a constant flowrate of 1 ml min^−1^. The heating program of the column oven started at 30°C, then increased at a rate of 30°C min^−1^ to 60°C for 2 min, at 2°C min^−1^ to 190°C, and at 5°C min^−1^ to 230°C, held for 10 min. The MS detector scanned within a mass range of m/z 30–450. Volatile compounds identification and quantification was performed as previously reported (D’Onofrio et al., 2017; Palai et al., 2022b). The monoterpenes geraniol, linalool, and α-terpineol derivatives were grouped following the aggregation based on their common biosynthetic derivation as proposed by D’Onofrio et al. (2017) with additional revisions suggested by Palai et al. (2022b). To avoid concentration or dilution effects caused by the different berry sizes induced by irrigation treatments, all VOC data are expressed as ng g^−1^ of berry DW.

### 2.5 RNA extraction and RNA-seq analysis

Transcriptome analysis was performed on 2020 samples, with a focus on the FI, RDI-1S, RDI-LS, and RDI-2S treatments. Berry sampling was done at the beginning of veraison (excluded RDI-2S), at mid-ripening (DOY 234), and at harvest. Three 50-berry replicates were randomly chosen for each irrigation treatments and immediately frozen in liquid nitrogen. The pedicels and seeds were carefully removed, and the remaining berries were crushed in liquid nitrogen. Total RNA was isolated from 0.3 g of berries powder using the Spectrum Plant Total RNA kit (Sigma-Aldrich, St. Louis, MO, USA) according to the manufacturer’s instructions. The quality and concentration of RNA were detected by agarose gel electrophoresis and by using a NanoDrop 2000 (Thermo Fisher Scientific, MA, USA). The RNA-seq analysis was performed by IGA Tech (IGA Technology Services Srl, Udine, Italy), sequencing the libraries on paired-end 150-bp mode on NovaSeq 6000 (Illumina, San Diego, CA, USA), including base calling and demultiplexing, adapters masking, trimming, alignments on the reference genome (12xCHR v2.1), quality control (statistics on strandness of reads, on gene body coverage, on read distribution, and on insert size), and the pair-wise differential expression analysis. Gene functional annotations were obtained from the Integrape grapevine reference catalogue (Navarro-Payá et al., 2021).

### 2.6 Experimental design and statistical analysis

Significant differences between treatments were determined by one-way analysis of variance (ANOVA, p ≤ 0.05), and means were separated by Tukey’s honestly significant difference (HSD). Principal component analysis (PCA) was performed over the free/glycosylated aroma compounds dataset (2 years, six irrigation treatments, and three replicates). All statistical analysis and the heatmaps representing log2 fold change (Log2FC) of the transcripts level between treatments (RDI/FI) were performed using the genomics and GWAS package of JMP Pro 17 (SAS Institute Inc., Drive, Cary, NC, USA).

## 3 Results

### 3.1 Vine water status and berry characteristics

The minimum Ψ_stem_ values of FI ranged between −0.50 and −0.60 MPa in 2019 and between −0.45 and −0.63 MPa in 2020. Before veraison, the lowest values of Ψ_stem_ were measured in RDI-1S vines (−1.85 and −1.50 MPa in 2019 and 2020, respectively), whereas after veraison, they were measured in RDI-2S vines (−2.00 and −1.60 MPa in 2019 and 2020, respectively) (**Table 1**). The RDI-LS vines experienced water deficit only during the lag phase, reaching minimum Ψ_stem_ values of −1.65 and −1.40 MPa in 2019 and 2020, respectively. The dWSI was higher in RDI-1S (1.47 and 1.42 MPa in 2019 and 2020, respectively) and RDI-2S (1.64 and 1.17 MPa in 2019 and 2020, respectively) before and after veraison, respectively (**Table 2**).

**Table 1:** Minimum stem water potential (Ψ_stem_) and daily water stress integral (dWSI) of Sangiovese grapevines (*Vitis vinifera* L.) measured in 2019 and 2020 from berry pea size to lag phase (PS-L), from lag phase to beginning of veraison (L-V), from beginning of veraison to harvest (V-H), and from pea size to harvest (PS-H).

Year	Irrigation treatment	Minimum Ψ_stem_ (MPa)			dWSI (MPa)			
		PS-L	L-V	V-H	PS-L	L-V	V-H	PS-H
2019	FI	-0.60	-0.50	-0.50	0.44	0.44	0.38	0.41
	RDI-1S	-1.70	-1.85	-0.60	1.07	1.47	0.65	0.90
	RDI-1M	-1.10	-1.60	-0.55	0.81	1.24	0.53	0.70
	RDI-LS	-0.75	-1.65	-0.55	0.70	1.20	0.61	0.66
	RDI-2S	-0.55	-1.00	-2.00	0.46	0.53	1.64	0.94
	RDI-2M	-0.85	-1.50	-1.95	0.46	0.48	1.16	0.75
2020	FI	-0.45	-0.45	-0.63	0.32	0.35	0.50	0.40
	RDI-1S	-1.50	-1.50	-0.85	1.03	1.42	0.65	0.86
	RDI-1M	-1.20	-1.15	-0.65	0.76	1.07	0.60	0.69
	RDI-LS	-0.45	-1.40	-0.65	0.50	0.98	0.52	0.51
	RDI-2S	-0.43	-0.45	-1.60	0.31	0.36	1.17	0.78
	RDI-2M	-0.47	-0.50	-1.30	0.32	0.36	0.81	0.63

Values are means of five replicate vines per treatment. (FI, full irrigation from berry pea size to harvest; RDI-1S and RDI-1M, severe and moderate water deficit applied from berry pea size to veraison; RDI-LS, water deficit applied during lag phase; RDI-2S and RDI-2M, severe and moderate water deficit applied from veraison to harvest).

**Table 2:** Free VOCs measured at harvest in berries of Sangiovese grapevines (*Vitis vinifera* L.) subjected to six irrigation regimes (FI, full irrigation from berry pea size to harvest; RDI-1S and RDI-1M, severe and moderate water deficit applied from berry pea size to veraison; RDI-LS, water deficit applied during lag phase; RDI-2S and RDI-2M, severe and moderate water deficit applied from veraison to harvest) in 2019 and 2020.

	2019							2020						
	FI	RDI-1S	RDI-1M	RDI-LS	RDI-2S	RDI-2M	*I*	FI	RDI-1S	RDI-1M	RDI-LS	RDI-2S	RDI-2M	*I*
isoamyl alcohol	4.6 ab	2.4 b	4.9 ab	9.6 a	7.9 ab	6.1 ab	*	2.8	2.2	2.6	7.6	5.7	5.9	n.s.
1-pentanol	4.5 ab	2.4 b	5.4 ab	9.4 a	7.9 ab	5.7 ab	*	2.4	2.3	2.5	7.7	5.4	5.9	n.s.
3-methyl-2-buten-1-ol	36.2 b	7.1 d	49.6 a	11.5 d	12.6 d	24.4 c	***	21.5 bc	25.1 ab	11.9 cd	34.0 a	7.2 d	15.8 bcd	***
1-hexanol	306.3 ab	243.0 b	405.0 a	406.1 a	351.7 ab	358.8 ab	*	241.3	215.8	320.1	320.2	315.5	287.4	n.s.
*trans*-3-hexenol	7.2 b	7.4 b	12.8 a	13.1 a	10.4 ab	8.8 ab	**	7.7	8.3	8.6	9.6	9.6	8.0	n.s.
*cis*-3-hexenol	50.5 ab	25.4 b	64.5 a	33.5 b	43.8 ab	68.3 a	**	45.0	45.1	74.7	49.0	42.1	48.9	n.s.
*trans*-2-hexenol	1037	815.7	1154	995.5	975.0	1020	n.s.	706.5 bc	569.8 c	1039 a	842.7 abc	743.9 bc	957.4 ab	**
1-octen-3-ol	12.7 bc	3.5 d	28.2 a	6.2 d	7.8 cd	17.4 b	***	14.3 ab	15.9 a	4.7 c	22.2 a	4.9 b	6.8 bc	***
ethylhexanol	2.8	2.4	3.5	2.9	3.4	3.7	n.s.	2.8	3.4	2.7	4.4	2.7	2.6	n.s.
**Total aliphatic alcohols**	**1461**	**1109**	**1727**	**1487**	**1421**	**1513**		**1044**	**887.9**	**1467**	**1297**	**1137**	**1338**	
benzaldehyde	1.6	1.5	1.8	15	1.8	1.8	n.s.	1.5	1.2	1.4	1.6	1.3	1.8	n.s.
acetophenone	2.8 b	5.4 a	3.8 b	3.6 b	4.0 b	3.5 b	n.s.	4.2 c	5.3 abc	5.7 ab	6.1 a	4.6 bc	6.2 a	n.s.
2,5-dimethyl-benzaldehyde	1.1 b	0.7 bc	2.9 a	0.3 c	0.9 b	0.8 b	**	0.8	1.1	1.5	0.2	0.2	1.3	n.s.
benzyl alcohol	79.9 c	101.9 bc	67.5 c	193.6 ab	262.8 a	118.3 bc	**	119.0 ab	40.3 b	54.6 b	112.8 ab	134.2 ab	204.3 a	**
2-phenylethanol	445.3 b	217.7 c	490.3 b	590.3 ab	483.5 b	683.7 a	***	423.5 abc	157.6 c	268.8 bc	615.5 a	629.6 a	551.2 ab	**
benzoic acid, 4-ethoxy-, ethyl ester	1.8	2.4	3.1	2.3	2.7	3.6	n.s.	1.7	2.0	1.9	2.8	2.4	2.5	n.s.
**Total benzene derivatives**	**532.5**	**329.6**	**569.4**	**791.5**	**755.6**	**811.6**		**550.7**	**207.5**	**333.9**	**739.0**	**772.4**	**767.2**	
guaiacol	3.3 d	3.6 d	5.8 cd	9.0 a	8.3 ab	6.5 bc	***	4.41 bcd	3.3 d	3.9 cd	6.7 abc	7.9 a	7.2 ab	**
4-vinylguaiacol	68.8	37.0	50.5	109.7	113.4	84.7	n.s.	103.3 ab	30.9 b	149.3 a	97.4 ab	113.4 a	117.9 a	**
phenol, 2,6-dimethoxy	13.5 c	17.0 bc	17.2 bc	24.1 ab	29.6 a	20.2 abc	**	16.23	13.6	19.8	21.4	27.0	22.0	n.s.
**Total phenols**	**85.7**	**57.6**	**73.5**	**142.8**	**151.4**	**111.3**		**123.95**	**47.8**	**173.0**	**125.5**	**148.3**	**147.1**	
vanillin	3.7 cd	3.0 d	7.1 a	4.3 bcd	4.9 bc	5.6 ab	***	3.53 ab	2.6 b	7.2 a	5.0 ab	3.6 ab	5.4 ab	*
methyl vanillate	94.6	115.3	133.2	114.9	122.2	143.8	n.s.	68.66 bc	56.1 c	101.1 ab	103.1 ab	111.4 a	101.1 ab	**
acetovanillone	33.8 ab	17.8 b	33.7 ab	82.1 ab	120.7 a	86.8 ab	*	56.93 ab	14.3 b	44.6 ab	70.3 0ab	87.6 a	83.5 a	**
zingerone	6.8 ab	4.0 b	8.4 ab	10.7 ab	9.3 ab	12.7 a	*	7.95 ab	3.8 b	9.0 ab	10.0 ab	11.5 a	11.3 a	*
homovanillic alcohol	8.5	7.8	9.2	15.4	15.0	12.2	n.s.	9.47 b	6.2 b	38.6 a	13.7 b	16.2 b	19.7 b	**
homovanillic acid	6.7 bc	5.8 c	11.3 a	5.8 c	5.6 c	9.6 ab	**	7.22	4.7	10.5	8.0	6.9	7.9	n.s.
acetosyringone	5.2	6.3	6.4	10.1	16.9	10.6	n.s.	10.55	6.0	13.0	11.5	11.9	14.7	n.s.
**Total vanillins**	**159.3**	**160.2**	**209.4**	**243.3**	**294.6**	**281.36**		**164.34**	**93.8**	**224.0**	**221.4**	**249.1**	**243.6**	
α-terpineol ^T^	0.5	0.4	0.6	0.3	0.5	0.4	n.s.	0.2	0.3	0.6	0.4	0.4	0.4	n.s.
(*E*)-pyranoid linalool ox. C ^L^	2.8 bc	1.0 c	3.2 bc	5.9 a	3.4 abc	4.0 ab	**	2.5 a	0.6 b	1.0 b	2.2 a	3.1 a	3.0 a	***
(*Z*)-pyranoid linalool ox. D ^L^	3.1 b	1.2 b	4.6 ab	7.4 a	3.7 ab	4.4 ab	**	3.2 ab	0.5 c	1.0 bc	3.0 ab	3.2 ab	4.0 a	**
citronellol ^G^	5.2 b	4.1 b	9.2 a	5.3 b	4.8 b	8.7 a	***	4.5 ab	2.8 b	2.7 b	6.6 a	3.1 b	3.6 ab	*
nerol ^G^	7.6 bc	6.7 c	13.9 a	12.8 a	10.5 ab	12.8 a	***	7.4 abc	5.1 c	5.3 bc	9.6 a	8.1 ab	7.5 abc	**
geraniol ^G^	68.2	77.6	75.1	79.5	78.1	97.2	n.s.	33.5 b	22.8 b	36.3 b	51.2 a	53.3 a	62.2 a	***
2,6-dimethyl-3,7-octadiene-2,6-diol 1 ^L^	21.4 a	2.8 d	16.0 ab	5.2 bcd	3.0 cd	14.6 abc	**	5.9 ab	3.2 b	5.1 ab	7.5 a	2.9 b	5.4 ab	*
2,3-pinanediol	2.3	0.6	3.0	2.3	2.1	4.1	n.s.	2.1	0.9	0.6	1.7	1.7	2.7	n.s.
7-OH-geraniol ^G^	18.8 b	3.5 c	30.4 a	5.0 c	4.9 c	28.2 a	***	10.3 ab	6.6 ab	5.2 b	12.5 a	5.2 b	6.4 ab	*
geranic acid ^G^	113.8 b	116.4 b	144.4 ab	151.2 ab	183.7 a	164. ab	**	72.8 bc	48.3 c	69.1 bc	105.7 ab	134.8 a	122.2 a	***
**Total monoterpenes**	**243.6**	**213.8**	**300.5**	**275.0**	**294.6**	**338.7**		**142.5**	**90.3**	**126.1**	**200.3**	**215.9**	**217.5**	
hexanoic acid	167.1	177.2	173.8	168.4	173.6	232.6	n.s.	88.7 bc	63.5 c	141.9 ab	171.8 a	195.0 a	163.6 a	***
hexanoic acid, 2-ethyl-	79.9 c	101.9 c	67.5 c	241.9 ab	328.4 a	118.3 bc	***	119.0 ab	40.3 b	54.6 b	112.8 ab	134.2 ab	204.3 a	**
(*E*)-2-hexenoic acid	445.3 c	217.7 d	490.3 bc	737.8 a	604.3 abc	683.7 ab	***	423.5 abc	157.6 c	268.8 bc	615.5 a	629.6 a	551.2 ab	**
nonanoic acid	14.6	19.7	19.6	11.8	10.8	21.4	n.s.	10.7 b	14.5 b	26.1 a	14.5 b	12.7 b	11.4 b	**
*n*-decanoic acid	4.0	3.1	5.2	3.9	3.8	4.3	n.s.	2.6	4.3	4.6	4.9	2.6	2.6	n.s.
myristic acid	14.5 abc	12.4 bc	20.0 a	16.5 ab	8.5 c	15.9 ab	**	10.8 ab	9.8 b	12.6 ab	20.0 a	12.0 ab	11.3 ab	*
pentadecanoic acid	26.8 a	2.8 c	20.0 a	5.2 bc	3.0 c	18.3 ab	**	5.9 ab	3.2 ab	5.1 ab	7.5 a	2.3 b	5.4 ab	*
**Total carboxylic acids**	**752.1**	**534.8**	**796.4**	**1186**	**1133**	**1094**		**661.3**	**293.1**	**513.7**	**946.9**	**988.6**	**949.7**	
methyl palmitoleate	6.3	6.6	8.9	6.9	3.5	7.7	n.s.	6.3	8.1	4.8	9.9	6.4	4.6	n.s.
methyl stearate	79.9	40.7	79.1	74.3	90.3	70.5	n.s.	40.7	51.0	41.5	65.5	40.4	55.7	n.s.
*trans*-13-octadecenoic acid methyl ester	3.6	3.5	5.3	3.6	3.9	4.7	n.s.	2.5	2.3	3.3	3.2	3.8	3.6	n.s.
methyl linoleate	51.8 ab	37.0 b	50.5 ab	109.7 ab	113.4 a	84.7 ab	*	103.3 ab	30.9 b	128.0 a	92.4 ab	113.4 ab	147.3 a	*
methyl linolenate	131.9 ab	212.1 a	131.6 ab	171.2 ab	92.9 b	166.9 ab	**	142.1	188.0	139.9	200.8	172.3	129.4	n.s.
**Total esters**	**273.4**	**299.8**	**275.5**	**365.7**	**304.0**	**334.4**		**295.0**	**280.3**	**317.5**	**371.8**	**336.2**	**340.6**	
3-hydroxy-7,8-dihydro-β-ionol	1.7	0.6	2.4	2.3	2.0	2.9	n.s.	1.2	0.5	1.0	1.3	2.0	1.4	n.s.
(*E*)-2-hexenal	9.2 b	4.8 b	16.6 a	5.0 b	5.6 b	18.2 a	***	15.8	11.7	17.4	17.5	11.1	17.2	n.s.
benzothiazole	8.3 b	17.2 a	15.6 a	18.1 a	22.1 a	17.1 a	**	10.2 b	17.8 a	21.7 a	18.6 a	18.5 a	16.3 ab	**
4-methyl-5-thiazoleethanol	97.4 ab	101.7 ab	78.0 b	84.4 ab	107.9 a	45.3 c	***	14.4 c	29.5 bc	53.7 a	22.3 bc	41.7 ab	31.2 bc	**
2,3-dihydrobenzofuran	19.1	8.4	11.1	13.4	17.0	14.6	n.s.	11.5 ab	6.0 b	15.9 a	13.3 ab	13.3 ab	18.4 a	**
**Total others**	**135.8**	**132.7**	**123.8**	**123.2**	**154.6**	**98.2**		**53.0**	**65.5**	**109.7**	**72.9**	**86.5**	**84.5**	
**Total free VOCs**	**3644**	**2838**	**4076**	**4615**	**4508**	**4583**		**3035**	**1966**	**3265**	**3975**	**3934**	**4089**	

Values (ng g^−1^ of berry DW) are means of three replicates for each irrigation treatment. Different letters indicate honest significant differences between irrigation treatments after analysis of variance (ANOVA) within each year. I, irrigation; *p < 0.05; **p < 0.01; ***p < 0.001; n.s. (not significant). The superscript letter on monoterpenes indicates the biosynthetic origin, where G is geraniol, L is linalool and T is α-terpineol.

Berry FW at harvest ranged between 1.48 g (RDI-1S) and 2.54 g (FI), and berries from all RDI vines showed lower FW values than FI vines (**Supplementary Figure S1**, Palai et al., 2022a). All treatments reached similar sugar concentrations, since harvest date was decided based on that parameter. The berries from both moderate deficit irrigated vines (RDI-1M and RDI-2M) reached that threshold faster and earlier than the other treatments (**Supplementary Figure S1**, Palai et al., 2022a). At harvest, values of TA of RDI-1 berries were the lowest, whereas the TA of RDI-LS berries reached the highest values (6.37 and 6.90 g L^−1^ tartrate, in 2019 and 2020, respectively) even if not statistically different with respect to FI (**Supplementary Figure S1**, Palai et al., 2022a). The skin to pulp ratio and the specific skin weight (SSW) showed different patterns among treatments at veraison and harvest (**Supplementary Figure S2**). Berries from RDI-1S, RDI-1M, and RDI-LS vines showed higher values of skin to pulp ratio at veraison, which were statistically significant in 2020. At harvest, the highest skin to pulp ratio and SSW were measured in RDI-2S berries in both years (**Supplementary Figure S2**). Since aroma compounds are mainly located in the skin, these results allowed us to repute a negligible indirect effect of water deficit in determining differences in berry VOCs across irrigation treatments. Indeed, the lowest VOC concentration at harvest was measured in RDI-2 berries, which had the highest values of skin to pulp ratio and SSW (**Supplementary Figure S2**).

### 3.2 Berry VOCs

**Figure 1:**
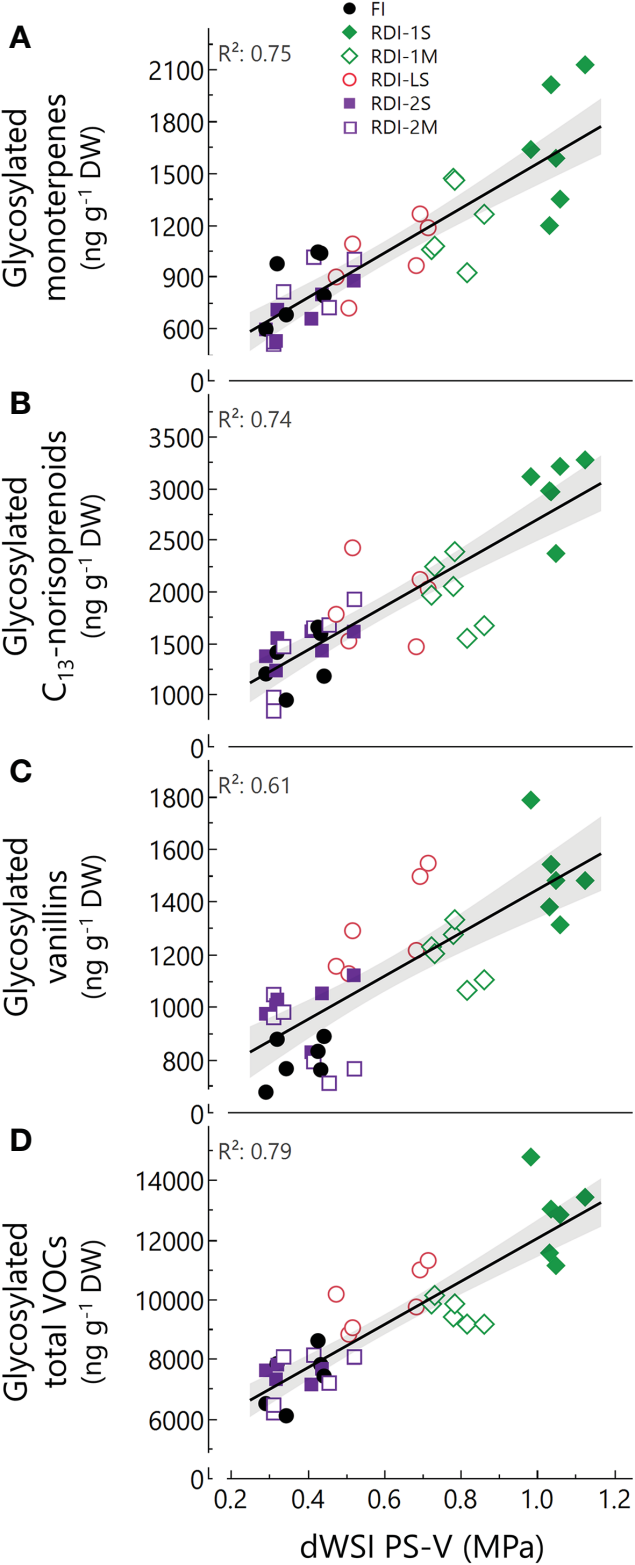
The relationship between total glycosylated monoterpenes, total glycosylated C_13_-norisoprenoids, total glycosylated vanillins, total glycosylated VOCs, and daily water stress integral (dWSI) from pea size (PS) to the beginning of veraison (V) in berries of Sangiovese (*Vitis vinifera* L.) grapevines subjected to six different irrigation regimes (FI, full irrigation from berry pea size to harvest; RDI-1S and RDI-1M, severe and moderate water deficit applied from berry pea size to beginning of veraison; RDI-LS, water deficit applied during lag phase; RDI-2S and RDI-2M, severe and moderate water deficit applied from beginning of veraison to harvest) in 2019 and 2020. Equations: **(A)** y=257.4+1259x; **(B)** y=584.3+2111x; **(C)** y=622+822.6x; **(D)** y=4801+7230x. Each symbol represents one replicate. Confidence interval (95%) in grey.

The highest total glycosylated VOC concentration was measured in RDI-1S berries (12,017 and 12,545 ng g^−1^ of berry DW in 2019 and 2020, respectively), while the lowest one was measured in RDI-2S (7,108 ng g^−1^ of berry DW) and in FI berries (6286 ng g^−1^ of berry DW) in 2019 and 2020, respectively (**Table 3**). Despite the short period of water stress experienced (14 and 13 days in 2019 and 2020, respectively), RDI-LS berries showed the highest concentrations of total glycosylated VOCs after RDI-1S (10,169 and 9,508 ng g^−1^ of berry DW in 2019 and 2020, respectively). Glycosylated linalool and its derived compounds were the most abundant monoterpenes and reached the highest concentrations under RDI-1S treatment (+107 and +55% than FI, in 2019 and 2020, respectively) (**Table 3**). Similarly, glycosylated geraniol and its derived compounds increased by 136% and 125% than FI (2019 and 2020, respectively), and α-terpineol and its derived compounds (+68 and +111% than FI, in 2019 and 2020, respectively). Water deficit applied after veraison was detrimental for the accumulation of total glycosylated monoterpenes (−11% and −15% in 2019 and 2020, respectively, average between RDI-2S and RDI-2M). The RDI-1S, RDI-1M, and RDI-LS berries had the highest glycosylated C_13_-norisoprenoids concentration (**Table 3**). In particular, the 3-oxo-α-ionol and damascenone reached values up to more than twofold in RDI-1S berries with respect to FI ones in both years. 2-Phenylethanol was quantitatively the most important glycosylated benzene derivative, and it was particularly concentrated in RDI-1S berries in both years. The total glycosylated phenols increased with respect to FI within all treatments, whereas vanillins were mainly enhanced in RDI-1S followed by RDI-LS and RDI-1M berries, in both years (**Table 3**). **Figure 1** shows a general relationship between the water stress experienced by vines from pea size to veraison and the concentration of glycosylated VOCs at harvest. A positive linear relationship was observed between dWSI and the amount of total glycosylated VOCs (R^2^ = 0.79), monoterpenes (R^2^ = 0.75), C_13_-norisoprenoids (R^2^ = 0.74), and vanillins (R^2^ = 0.61).

**Table 3:** Glycosylated VOCs measured at harvest in berries of Sangiovese grapevines (*Vitis vinifera* L.) subjected to six irrigation regimes (FI, full irrigation from berry pea size to harvest; RDI-1S and RDI-1M, severe and moderate water deficit applied from berry pea size to veraison; RDI-LS, water deficit applied during lag-phase; RDI-2S and RDI-2M, severe and moderate water deficit applied from veraison to harvest) in 2019 and 2020.

	2019							2020						
	FI	RDI-1S	RDI-1M	RDI-LS	RDI-2S	RDI-2M	*I*	FI	RDI-1S	RDI-1M	RDI-LS	RDI-2S	RDI-2M	*I*
isoamyl alcohol	89.8 b	141.0 ab	84.0 b	165.8 a	111.6 ab	101.5 ab	**	101.0	144.4	113.5	136.3	156.4	135.1	n.s.
1-pentanol	27.0 c	70.6 a	45.8 abc	63.8 ab	39.4 bc	40.1 bc	**	28.4 b	61.1 a	40.5 b	37.3 b	30.9 b	35.3 b	**
3-methyl-2-buten-1-ol	112.0 b	160.7 a	128.5 ab	167.3 a	95.1 b	84.2 b	**	76.6 b	126.8 a	111.3 ab	92.6 ab	74.8 b	88.6 ab	*
1-hexanol	103.0 c	163.8 ab	105.1 c	187.6 a	102.6 c	116.9 bc	**	99.3	145.9	117.1	118.6	122.7	105.0	n.s.
*cis*-3-hexenol	1.7 c	2.7 ab	1.7 c	3.3 a	1.7 c	2.0 bc	**	1.6	2.1	1.7	2.0	1.9	1.9	n.s.
*trans*-2-hexenol	20.9 ab	29.6 a	19.4 b	26.6 ab	24.7 ab	25.7 ab	*	18.7	29.6	26.0	23.4	23.8	23.0	n.s.
1-octen-3-ol	4.8	9.8	8.7	11.3	9.2	9.5	n.s	3.8	6.7	4.1	4.4	9.1	6.4	n.s.
1-octanol	5.1 b	8.4 a	4.0 b	7.4 a	2.6 c	4.7 b	***	5.7	8.6	8.2	7.8	6.2	5.6	n.s.
(*E*)-2-octen-1-ol	3.7 bc	5.2 a	3.3 c	4.9 ab	3.0 c	3.6 bc	**	3.7 c	6.3 a	4.3 bc	5.5 ab	4.6 bc	3.5 c	**
4-methyl-3-pentenol	49.2 b	64.6 b	71.7 ab	109.9 a	63.4 b	77.3 ab	**	69.7 ab	93.6 a	62.1 b	67.1 ab	72.9 ab	61.1 b	*
**Total aliphatic alcohols**	**417.0**	**656.4**	**472.1**	**747.8**	**453.0**	**465.5**		**408.5**	**624.9**	**488.9**	**495.2**	**503.3**	**465.5**	
benzaldehyde	7.7 bc	13.1 ab	4.6 c	15.0 ab	16.1 a	10.6 abc	**	41.7	10.9	5.9	11.0	8.3	10.2	n.s.
methyl benzoate	0.2	0.2	0.2	0.2	0.2	0.2	n.s.	0.1	0.2	0.1	0.1	0.1	0.1	n.s.
acetophenone	1.2 b	1.9 a	1.3 b	1.5 ab	1.4 ab	1.3 b	**	1.1	1.8	1.3	1.1	1.1	1.0	n.s.
ethyl benzoate	0.4	0.7	0.5	0.4	0.3	0.4	n.s.	0.3	0.6	0.3	0.4	0.3	0.3	n.s.
methyl salicylate	31.0 b	63.7 ab	31.7 b	122.9 a	127.5 a	86.6 ab	**	72.9 b	62.1 b	59.6 b	160.4 a	79.9 b	93.7 ab	**
benzyl alcohol	49.3	61.1	47.8	47.7	38.1	53.4	n.s.	32.0 b	67.0 a	38.4 b	38.4 b	28.9 b	25.2 b	***
2-phenylethanol	2449 bc	2978 a	2228 cd	2732 ab	1928 d	1906 d	***	2177 c	3111 a	2587 abc	3017 ab	2328 bc	19772 c	**
benzenepropanol	0.3	0.2	0.4	0.3	0.3	0.4	n.s.	0.2	0.1	0.3	0.3	0.2	0.2	n.s.
2-(4-methoxyphenyl)-ethanol	34.4 ab	46.7 a	36.2 ab	29.6 b	24.1 b	31.4 b	**	17.8 bc	40.8 a	29.2 ab	24.0 bc	16.3 c	14.8 c	***
6-methoxy-3-methylbenzofuran	4.1	4.4	2.8	3.2	3.3	3.9	n.s.	6.5	4.9	3.1	4.4	2.9	2.7	n.s.
benzoic acid	2.0	4.1	2.0	1.5	2.2	1.9	n.s.	1.8 b	4.6 a	2.6 b	2.0 b	1.9 b	1.3 b	**
3′,5′-dimethoxyacetophenone	14.1	13.2	12.4	14.8	15.3	18.9	n.s.	10.2	17.1	12.6	15.8	13.5	14.0	n.s.
3,4-dimethoxybenzyl alcohol	206.2	272.6	159.5	178.2	160.3	199.3	n.s.	167.5	240.4	293.4	265.5	190.5	257.3	n.s.
2,3,4-trimethoxybenzyl alcohol	61.2	54.9	60.2	72.5	47.0	57.6	n.s.	45.1 ab	72.7 a	49.3 ab	61.1 ab	44.2 ab	38.8 b	*
**Total benzene derivatives**	**2861**	**3515**	**2588**	**3220**	**2364**	**2372**		**2574**	**3634**	**3083**	**3602**	**2716**	**2436**	
cinnamic acid	4.6 b	7.7 a	4.5 b	5.5 ab	4.6 b	5.0 ab	*	3.1 c	6.4 a	4.3 abc	5.8 ab	3.9 bc	3.0 c	**
guaiacol	7.3 c	11.6 ab	8.3 bc	14.8 a	10.6 bc	11.6 ab	**	7.0 b	13.2 a	9.8 b	9.1 b	9.1 b	9.6 b	**
phenoxyethanol	5.2 a	5.6 a	4.8 a	5.0 a	4.2 ab	2.5 b	**	2.9	6.3	4.5	3.8	2.9	2.5	n.s.
4-vinylguaiacol	726.4 b	1235 ab	1035 ab	1432 a	665.4 b	799.5 b	**	482.3 b	1636 a	1068 ab	1169 ab	660.8 b	604.2 b	**
syringol	15.6 b	39.4 b	19.3 b	86.7 a	37.1 b	82.8 a	***	16.9 c	61.9 a	34.4 bc	38.8 b	42.7 ab	49.5 ab	**
eugenol	5.7	9.5	7.4	7.5	8.5	4.3	n.s.	6.1 b	14.4 a	9.0 ab	9.4 ab	4.9 b	5.1 b	**
methoxyeugenol	6.8	5.2	4.6	8.7	9.3	7.0	n.s.	6.8	6.1	4.5	9.9	7.3	6.8	n.s.
γ-hydroxyeugenol	28.0 a	33.2 a	32.1 a	24.7 ab	32.0 a	15.7 b	**	13.4 b	35.1 a	18.2 b	20.1 b	15.4 b	12.6 b	**
phenol-3,4,5-trimethoxy	76.3 b	126.5 a	77.2 b	142.7 a	78.1 b	81.1 b	**	66.5 b	124.5 a	75.8 b	96.3 ab	69.7 b	71.6 b	**
coniferyl alcohol	22.3 b	14.8 b	28.3 b	36.9 b	102.8 a	55.3 b	**	5.4 b	47.5 a	29.2 ab	42.0 a	36.6 a	33.3 a	**
**Total phenols**	**898.2**	**1488**	**1221**	**1765**	**952.5**	**1064**		**610.4**	**1951**	**1257**	**1404**	**853.3**	**798.0**	
vanillin	41.4 b	75.8 a	45.8 b	60.5 ab	53.8 ab	49.7 b	**	29.0 b	80.0 a	43.6 b	43.9 b	36.6 b	43.4 b	**
methyl vanillate	206.2 ab	272.6 a	138.8 b	194.9 ab	230.2 ab	159.5 ab	*	167.5 ab	240.4 a	293.4 a	265.5 a	34.4 b	230.3 a	**
acetovanillone	158.9 c	380.1 ab	515.4 a	489.8 a	255.1 bc	150.4 c	***	205.6 b	417.3 a	225.6 b	309.9 ab	190.5 b	232.7 b	**
zingerone	56.9	52.0	56.7	74.4	68.7	40.0	n.s.	46.3	69.7	47.7	78.8	32.6	45.8	n.s.
homovanillic alcohol	241.5 bc	433.7 a	344.6 ab	451.1 a	216.2 c	215.7 c	***	239.1 bc	510.8 a	258.7 b	355.5 ab	59.5 c	279.7 b	***
3,4,5-trimethoxybenzyl-methyl-ether	44.8	142.0	19.4	22.7	32.2	32.2	n.s.	17.9 c	22.6 c	141.3 b	33.2 c	353.4 a	83.4 bc	***
homovanillic acid	3.5	26.3	1.5	4.2	48.1	1.8	n.s.	0.9	17.2	10.6	4.2	23.0	7.3	n.s.
acetosyringone	76.5 b	117.8 a	114.0 a	120.9 a	95.0 ab	75.0 b	**	66.1 b	134.3 a	76.6 b	98.8 ab	2.8 c	75.2 b	***
**Total vanillins**	**829.8**	**1500**	**1236**	**1418**	**999.3**	**724.4**		**772.3**	**1492**	**1097**	**1189**	**732**	**997**	
(*E*)-furanoid linalool ox. A ^L^	25.8 bc	50.5 a	38.8 ab	49.8 a	24.2 bc	18.5 c	***	30.3	22.6	20.2	32.2	18.6	22.7	n.s.
(*Z*)-furanoid linalool ox. B ^L^	16.9 bc	33.3 a	26.1 ab	32.1 a	16.1 bc	14.0 c	***	18.0	13.7	11.9	19.2	13.7	14.2	n.s.
linalool ^L^	7.5 bc	31.6 a	10.9 bc	12.0 bc	6.7 c	18.3 b	***	4.6 b	30.3 a	19.6 a	5.3 b	2.8 b	3.2 b	***
terpinen-4-ol ^T^	2.5 c	7.0 a	4.3 b	4.6 b	3.1 bc	4.4 b	***	2.0 c	6.3 a	4.4 b	1.5 c	1.7 c	1.7 c	***
hotrienol ^L^	2.8 c	44.4 a	24.4 ab	6.6 bc	5.2 bc	2.6 c	***	4.8 b	35.7 a	5.4 b	19.6 ab	1.8 b	3.6 b	**
carvomenthol	0.7	1.6	0.9	1.2	0.7	0.9	n.s.	0.7	1.2	0.9	0.7	0.5	0.5	n.s.
ocimenol	13.4 b	28.1 a	25.0 ab	21.7 ab	19.5 ab	18.7 ab	*	11.2 b	28.2 a	14.6 b	12.1 b	10.7 b	10.5 b	***
*p*-cymen-7-ol	4.4	5.8	4.1	4.6	3.9	4.2	n.s.	3.5 b	6.7 a	4.2 b	4.3 b	3.3 b	2.9 b	**
*p*-cymen-8-ol	5.6 c	8.7 bc	15.4 a	10.1 bc	9.3 bc	12.5 ab	**	4.0 c	10.7 a	8.3 ab	5.9 bc	3.7 c	3.8 c	***
α-terpineol ^T^	17.6 b	36.4 a	32.8 ab	28.0 ab	25.3 ab	24.3 ab	*	14.6 b	36.7 a	18.9 b	15.7 b	14.0 b	13.7 b	***
α-citral ^G^	3.9 c	10.5 a	5.6 bc	6.8 b	4.1 bc	4.9 bc	***	3.5 cd	7.0 a	5.3 b	4.2 c	2.5 d	2.6 d	***
(*E*)-pyranoid linalool ox. C ^L^	56.2 bc	91.0 a	82.7 ab	100.2 a	54.9 bc	42.6 bc	**	58.8	45.5	40.5	61.9	49.4	44.8	n.s.
(*Z*)-pyranoid linalool ox. D ^L^	26.6 bc	51.9 a	39.5 ab	50.6 a	23.7 bc	22.4 c	***	27.1	16.6	16.1	28.4	19.3	21.7	n.s.
citronellol ^G^	8.2 c	39.1 a	24.2 b	12.8 c	10.2 c	4.4 c	***	4.3 bc	13.6 a	10.0 ab	9.0 abc	3.0 c	3.8 bc	**
lilac alcohol A ^L^	16.0 d	45.9 a	27.0 bc	30.2 b	19.8 cd	19.6 cd	***	14.0 bc	30.2 a	23.0 ab	18.6 bc	13.0 bc	11.9 c	**
myrtenol ^T^	3.9 c	7.4 a	6.4 ab	6.3 ab	4.7 bc	4.9 bc	**	4.0 b	7.6 a	5.0 b	5.1 b	3.4 b	3.7 b	***
nerol ^G^	27.9 d	66.9 a	39.9 cd	58.3 ab	32.5 cd	44.6 bc	***	31.9 c	58.1 a	49.9 ab	39.2 bc	26.0 c	28.7 c	***
isogeraniol ^G^	2.6	4.6	3.4	4.4	3.1	3.4	n.s.	3.0 bc	5.0 a	4.0 ab	3.5 bc	2.3 c	2.4 c	**
lilac alcohol B ^L^	4.0 c	26.9 a	17.0 b	17.4 b	15.4 b	5.4 c	***	4.9 cd	13.5 ab	15.3 a	8.5 bc	3.3 cd	2.9 d	***
geraniol ^G^	64.3 b	135.7 a	72.2 b	117.0 a	67.2 b	101.6 ab	**	80.4 cd	151.5 a	110.2 b	100.3 bc	61.5 d	69.7 d	***
exo-2-hydroxycineole ^T^	34.1 c	68.2 a	55.4 ab	51.9 ab	41.9 bc	42.8 bc	**	34.7 b	72.5 a	42.6 b	41.6 b	31.8 b	28.1 b	***
2,6-dimethyl-3,7-octadiene-2,6-diol 1 ^L^	7.4 abc	18.2 a	15.8 ab	5.9 bc	2.9 c	3.0 c	**	3.6	4.3	3.4	3.0	2.2	2.3	n.s.
6,7-2OH-7-hydroxylinalool ^L^	3.9	8.1	5.7	2.4	3.4	1.1	n.s.	2.6	5.0	2.4	2.4	1.1	1.2	n.s.
limonene-10-ol	2.7	6.2	4.8	4.6	4.0	3.8	n.s.	2.5	6.6	4.3	5.1	3.2	2.7	n.s.
2,6-dimethyl-1,7-octadiene-3,6-diol 2 ^L^	6.5 b	12.3 a	10.5 ab	11.0 ab	8.6 ab	8.8 ab	*	6.7 b	13.7 a	8.2 b	8.4 b	6.2 b	5.8 b	***
2,3-pinanediol	31.9 bc	52.8 a	36.2 b	38.3 b	22.2 c	28.6 bc	***	13.5 c	53.9 a	37.1 ab	31.6 abc	20.8 bc	24.2 bc	**
OH-citronellol ^G^	0.7	1.8	1.2	0.5	0.3	0.1	n.s.	0	0.5	0	0	0.1	0	–
*trans*-8-hydroxylinalool ^L^	171.5 bc	289.8 a	260.0 ab	113.7 c	90.8 c	83.2 c	***	89.4 b	113.9 a	108.9 a	94.6 b	86.9 b	73.6 b	**
*cis*-8-hydroxylinalool ^L^	84.0 b	196.3 a	95.2 b	74.0 b	49.7 b	96.6 b	***	51.0 b	124.8 a	92.1 a	50.7 b	37.8 b	34.1 b	***
geranic acid ^G^	132.6 b	308.3 a	164.5 b	165.4 b	113.4 b	204.4 ab	**	128.1 bc	328.1 a	233.4 ab	179.1 bc	99.6 c	103.4 c	***
7-OH-α-terpineol ^T^	82.8 b	117.1 a	122.4 a	23.0 c	28.5 c	20.9 c	***	24.2 ab	44.1 a	28.3 ab	23.0 ab	20.3 b	19.5 b	*
2,6-dimethyl-6OH-2,7-octadienoic acid ^L^	83.0 bc	158.2 a	122.8 ab	62.9 c	56.2 c	52.7 c	**	43.4	88.0	70.3	63.5	58.7	50.2	n.s.
**Total monoterpenes**	**951.6**	**1964**	**1395**	**1128**	**771.7**	**917.8**		**724.9**	**1396**	**1019**	**897.9**	**623.2**	**614.0**	
1-(2,6,6-trimethyl-1,3-cyclohexadien-1-yl)ethanol	12.8 cd	22.8 a	19.3 ab	22.1 a	17.0 bc	11.9 d	***	11.2 ab	13.33 a	11.2 ab	12.72 a	11.49 ab	9.49 b	*
damascenone	34.1 c	70.0 a	50.9 bc	58.1 ab	32.5 c	39.8 bc	***	28.8 c	58.5 a	49.9 ab	38.8 bc	25.57 c	28.56 c	***
actinidol A	2.8 c	5.8 a	4.0 bc	4.4 ab	3.3 bc	3.9 bc	**	3.2 c	8.96 a	5.8 bc	6.96 ab	3.97 c	2.99 c	***
actinidol B	5.0 c	10.1 a	7.2 bc	7.8 ab	5.3 bc	5.3 bc	**	5.6 c	16.29 a	10.39 bc	12.53 ab	6.76 c	4.81 c	***
2,5,5,8a-tetramethyl-1,2,3,5,6,7,8,8a-octahydronaphtalen-1-ol	8.8 b	21.6 a	13.1 ab	9.7 b	9.5 b	14.3 ab	*	9.9 b	33.05 a	16.95 b	12.87 b	10.05 b	9.63 b	**
3,4-dihydro-3-oxo-α-ionol (I)	60.9 b	105.1 a	75.5 ab	78.0 ab	64.2 b	70.3 b	**	55.6 b	140.5 a	80. b	78.9 b	66.2 b	49.9 b	**
3,4-dihydro-3-oxo-α-ionol (II)	168.2 b	275.4 a	192.7 b	202.9 ab	160.6 b	178.1 b	**	141.2 b	334.2 a	203.5 ab	197.7 ab	164.6 b	122.9 b	**
3,4-dihydro-3-oxo-α-ionol (III)	208.8 b	319.6 a	229.9 ab	238.9 ab	191.1 b	215.7 b	*	172.3 b	381.4 a	233.1 ab	236.3 ab	202.7 ab	144.9 b	**
3-hydroxy-β-damascone	8.4	15.2	2.9	9.5	10.2	10.1	n.s.	6.2 b	14.5 a	6.8 b	9.4 ab	6.1 b	6.0 b	**
3-oxo-α-damascone	17.1	28.5	21.8	22.0	19.2	21.7	n.s.	17.6 b	35.7 a	19.8 b	21.8 b	16.1 b	16.3 b	**
4-hydroxy-β-ionol	5.4 b	16.7 a	6.0 b	5.8 b	6.9 b	7.3 b	*	5.2 b	18.5 a	8.5 ab	8.1 ab	5.8 b	5.1 b	**
3-oxo-α-ionol	809.9 b	1805 a	1257 ab	1093 b	924.6 b	1022 b	**	630.4 c	2138 a	1116 b	1122 b	746.2 bc	607.4 c	***
2,3-2OH-4-oxo-7,8-2OH-β-ionon	48.7 b	79.3 a	54.0 b	64.2 ab	54.8 b	50.0 b	**	44.8 b	83.6 a	53.3 ab	71.9 ab	55.1 ab	41.7 b	*
epimanool	9.7 b	19.2 a	10.9 b	11.6 ab	9.9 b	11.5 ab	*	8.1 b	21.9 a	11.4 b	11.8 b	10.1 b	8.2 b	**
3,9-dihydroxy-megastigman-5-en	15.4	28.4	18.2	14.7	12.8	21.3	n.s.	12.2 b	36.4 a	18.2 b	18.3 b	14.2 b	10.7 b	**
blumenol-C	2.5	4.2	3.1	4.0	3.5	3.7	n.s.	1.7	5.0	3.0	3.3	3.2	2.1	n.s.
3-hydroxy-7,8-dihydro-β-ionol	3.3 b	7.5 a	4.2 ab	4.7 ab	4.5 ab	4.2 ab	*	2.7 b	9.5 a	4.5 b	4.7 b	3.7 b	2.9 b	***
7,8-dihydrovomifoliol	65.0 ab	57.4 ab	82.1 a	38.4 b	36.4 b	74.3 a	**	39.3 b	95.9 a	73.8 ab	51.4 ab	43.2 b	34.6 b	**
**Total C_13_-norisprenoids**	**1487**	**2891**	**2052**	**1890**	**1567**	**1765**		**1196**	**3446**	**1927**	**1919**	**1395**	**1108**	
**Total glycosylated VOCs**	**7445**	**12017**	**8966**	**10169**	**7108**	**7310**		**6286**	**12545**	**8873**	**9508**	**6824**	**6420**	

Full values (ng g^−1^ of berry DW) are means of three replicates for each irrigation treatment. Different letters indicate honest significant differences between irrigation treatments after analysis of variance (ANOVA) within each year. I, irrigation; *p < 0.05; **p < 0.01; ***p < 0.001; n.s. (not significant). The superscript letter on monoterpenes indicates the biosynthetic origin, where G is geraniol, L is linalool, and T is α-terpineol.

The highest total free VOC concentrations were measured in RDI-LS and in both RDI-2 treatments, whereas the lowest ones were measured in RDI-1S berries (**Table 2**). Among monoterpenes, free geranic acid was quantitatively the most important, especially in RDI-2 berries, which showed the highest concentrations. Similar results were observed for free geraniol as well, despite only in 2020, whereas free nerol had the highest values in berries from RDI-1M and RDI-LS vines in 2019 and 2020, respectively. 2-Phenylethanol and benzyl alcohol were the most represented free benzene derivatives, significantly affected by RDI treatments. Among carboxylic acids, the (*E*)-2-hexenoic acid was quantitatively the most important and showed the highest concentrations in RDI-LS and in both RDI-2 treatments.

**Figure 2:**
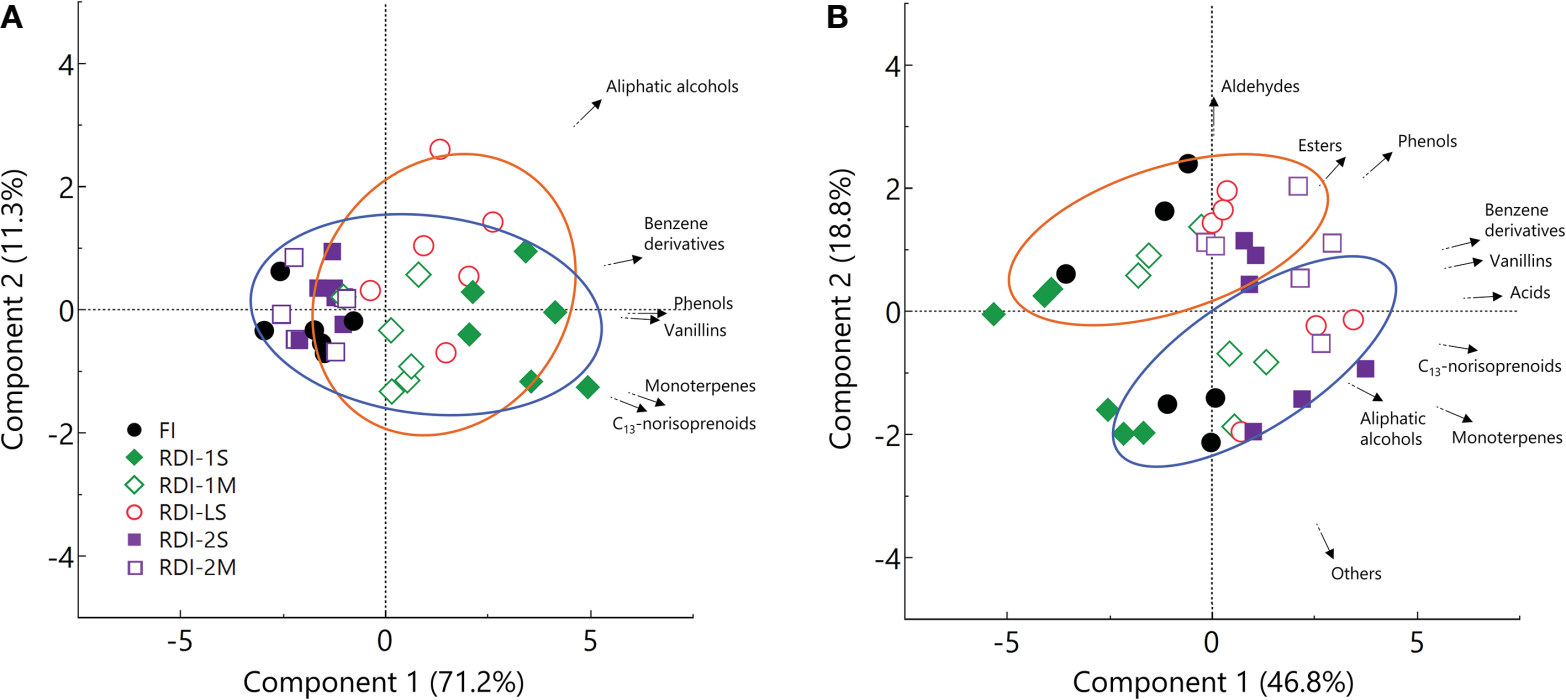
Principal component analysis performed on glycosylated **(A)** and free **(B)** VOCs from the 2019 (blue centroid) and 2020 (orange centroid) dataset, of berries of Sangiovese (*Vitis vinifera* L.) grapevines subjected to six different irrigation regimes (FI, full irrigation from berry pea size to harvest; RDI-1S and RDI-1M, severe and moderate water deficit applied from berry pea size to beginning of veraison; RDI-LS, water deficit applied during lag phase; RDI-2S and RDI-2M, severe and moderate water deficit applied from beginning of veraison to harvest). Each symbol represents one replicate.

The effect of irrigation regimes on berry total VOCs (free + glycosylated) was similar to what was reported for the glycosylated fraction, since they contributed for approximately 70%. The PCA provided a general overview of the distribution of free and glycosylated VOCs over the whole 2-year dataset (**Figure 2**). Considering glycosylated VOCs, the principal component (PC1) had an eigenvalue of 4.27 and described the higher part of the variability (71.2%) discriminating the irrigation regimes (**Figure 2A**), whereas PC2 described only the 11.3% of the total variance. The PC1 constructed over the free VOC dataset had an eigenvalue of 4.69 and described 46.8% of the total variation, explaining the variability induced by irrigation treatments (**Figure 2B**), whereas, contrary to what was observed for glycosylated VOCs, PC2 described 18.8% of the total variance discriminating the samples from the two experimental years.

### 3.3 Terpenoids and carotenoids pathway

**Figure 3:**
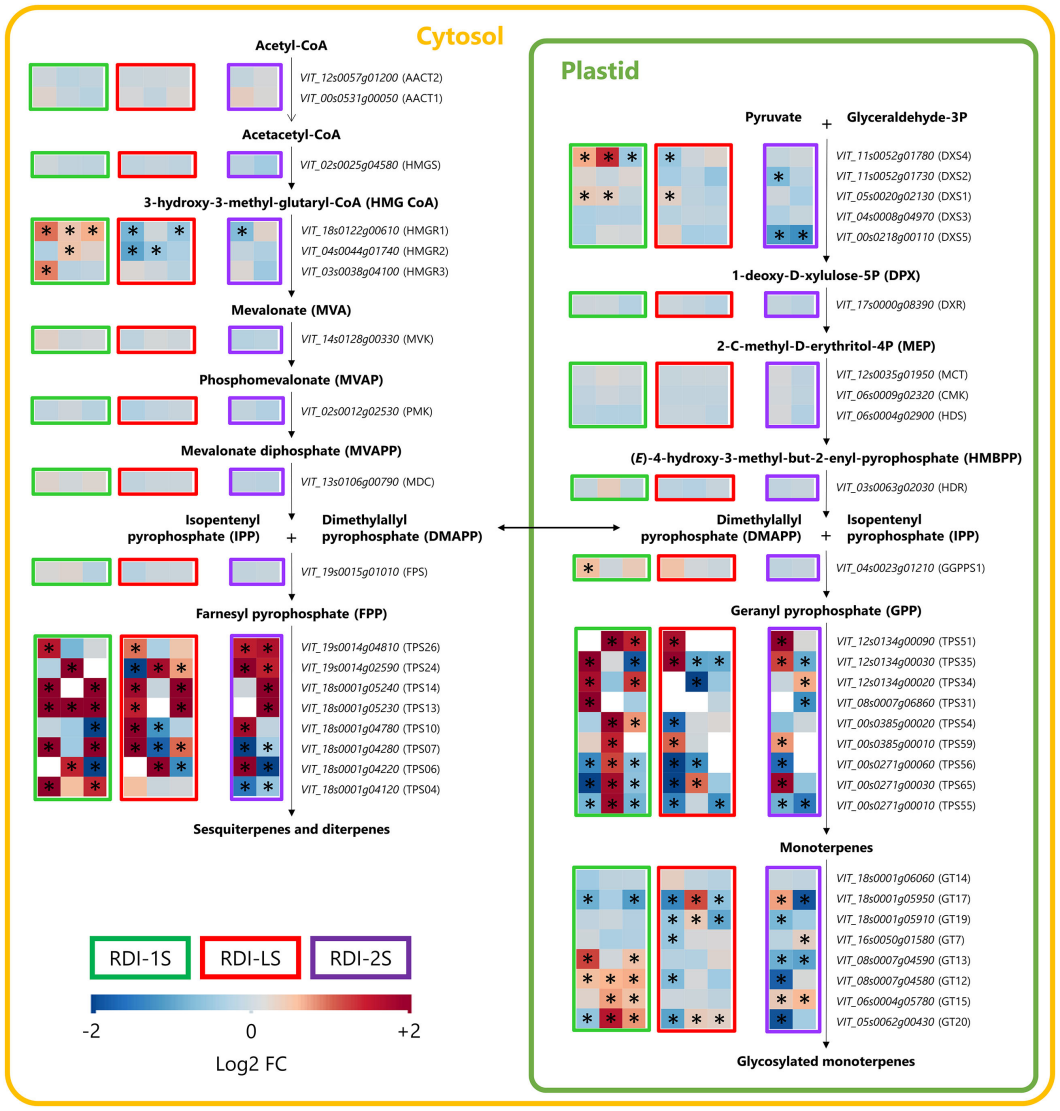
Modulation of MVA and MEP terpenes pathways in berries of Sangiovese grapevines (*Vitis vinifera* L.) subjected to different irrigation regimes: RDI-1S, severe water deficit applied from berry pea size to beginning of veraison (green block); RDI-LS, water deficit applied during lag phase (red block); RDI-2S, severe water deficit applied from beginning of veraison to harvest (purple block). Log2FC (RDI/FI) levels of differential gene expression are presented at the beginning of veraison (left box), mid-ripening (central box), and harvest (right box). White empty boxes indicate not detected genes. Asterisks indicate significant differences (p < 0.05) between treatments.

**Figure 4:**
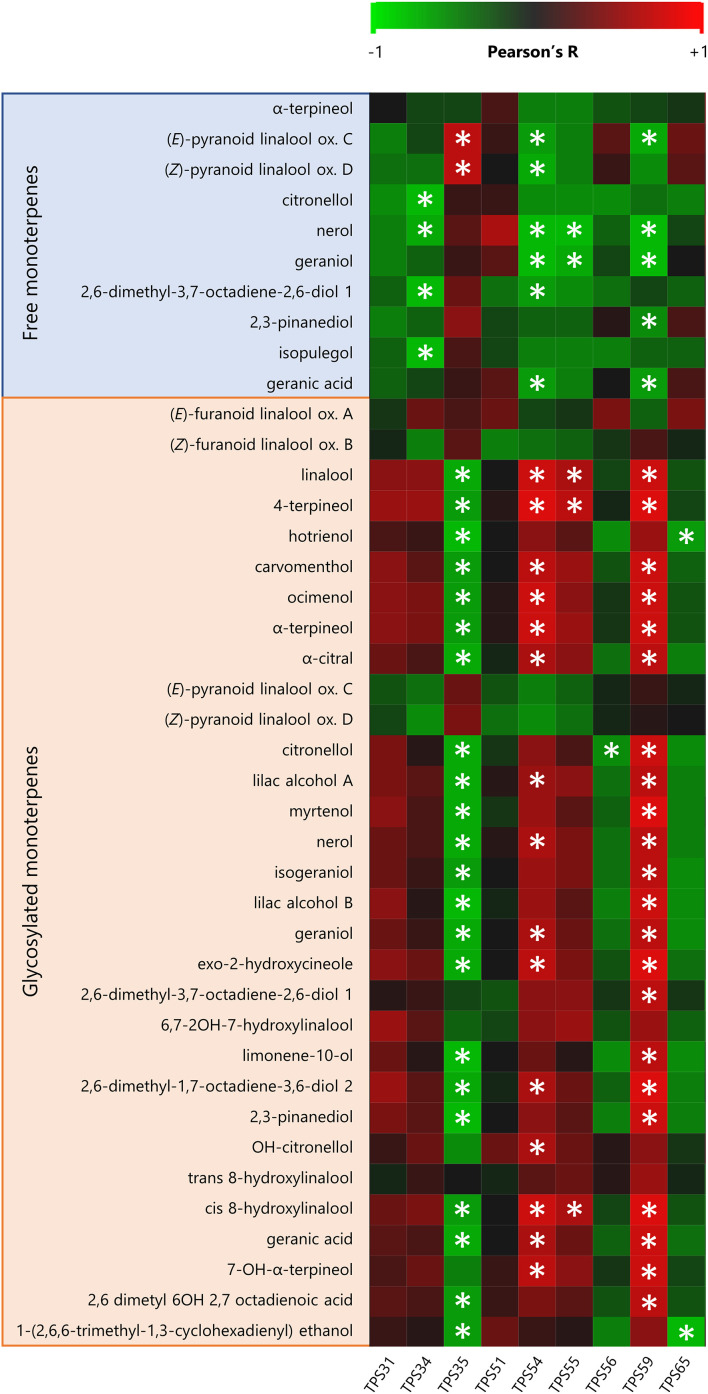
Pearson’s correlation heatmap between free and glycosylated berry monoterpenes and the relative TPSs expression in berries of Sangiovese grapevines (*Vitis vinifera* L.) subjected to different irrigation regimes (FI, full irrigation from berry pea size to harvest; RDI-1S and RDI-1M, severe and moderate water deficit applied from berry pea size to beginning of veraison; RDI-LS, water deficit applied during lag-phase; RDI-2S and RDI-2M, severe and moderate water deficit applied from beginning of veraison to harvest). Asterisks identify significant correlation (p<0.05).

**Figure 5:**
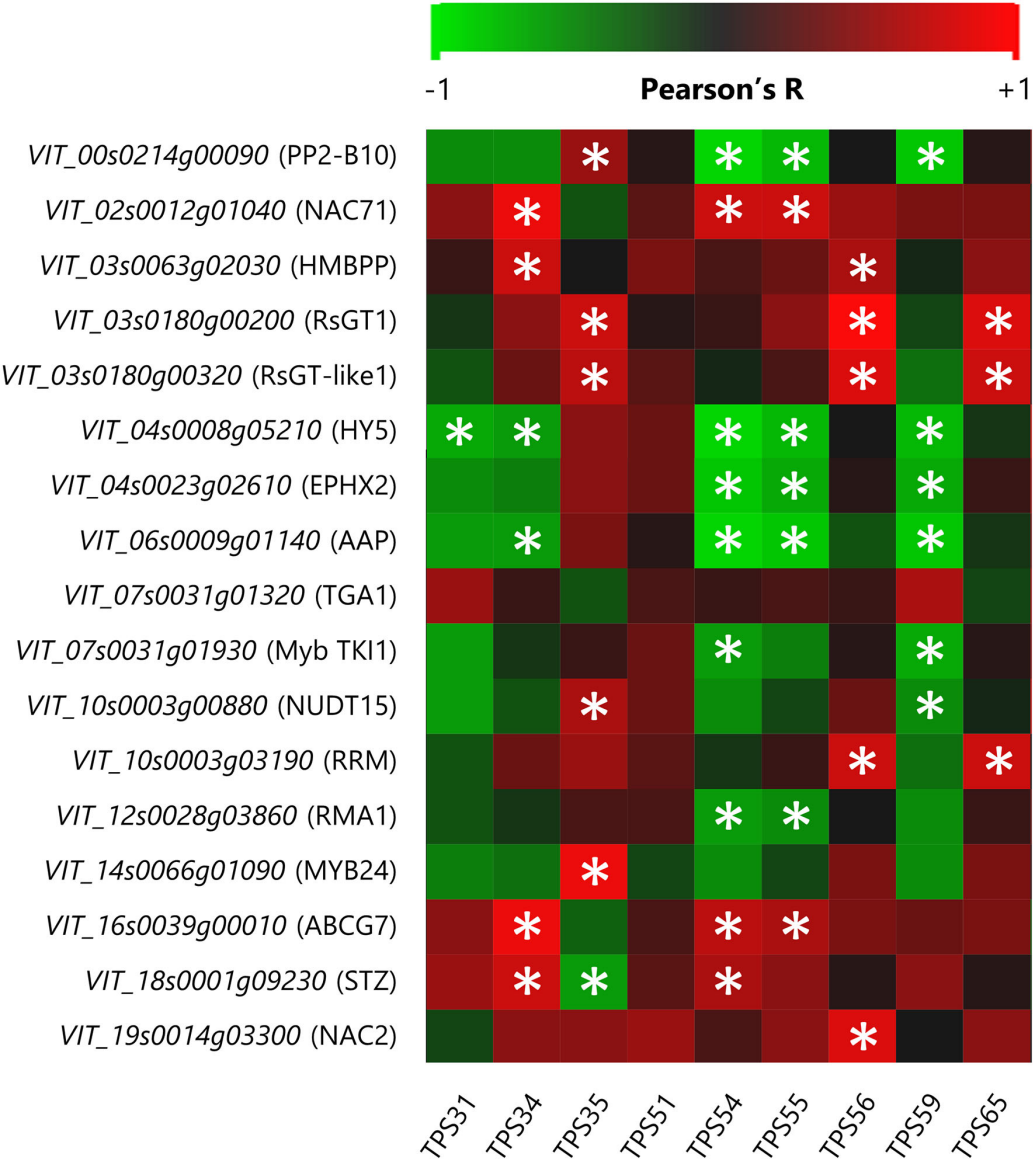
Pearson’s correlation heatmap between putative genes proposed for regulating the terpenes biosynthesis and monoterpenes synthases genes in berries of Sangiovese grapevines (*Vitis vinifera* L.) subjected to different irrigation regimes (FI, full irrigation from berry pea size to harvest; RDI-LS, water deficit applied during lag phase; RDI-1S and RDI-1M, severe and moderate water deficit applied from berry pea size to beginning of veraison; RDI-2S and RDI-2M, severe and moderate water deficit applied from beginning of veraison to harvest). Asterisks identify significant correlation (p<0.05).

Through the cytosolic mevalonate (MVA) pathway, the genes of the 3-hydroxy-3-methylglutaryl-CoA reductases (HMGRs) were upregulated in RDI-1S berries, even far after water stress had been released (**Figure 3**). On the contrary, RDI-LS and RDI-2S treatments downregulated their expression especially for HMGR1. The terpene synthase (TPS) 13, TPS14, TPS24, and TPS26 involved in the sesquiterpenes accumulation were overexpressed in all RDI treatments. In the plastidial methylerythritol phosphate pathway (MEP) pathway, the RDI-1S treatment significantly promoted the expression of 1-deoxy-d-xylulose 5-phosphate synthase (DXS) 1 and DXS4 until mid-ripening, whereas DXS5 was downregulated in RDI-2S berries (**Figure 3**). Downstream the MEP pathway, the geranylgeranyl pyrophosphate synthase (GGPPS) 1 was upregulated in RDI-1S berries at veraison. During ripening, all of the TPSs responsible for the monoterpenes biosynthesis were significantly overexpressed in RDI-1S berries. TPS35, TPS51, and TPS59 were promoted at veraison in RDI-LS berries, whereas TPS54, TPS56, and TPS65 were downregulated. The water deficit applied after veraison significantly reduced the expression of TPS31, TPS55, and TPS56, while TPS34, TPS51, TPS59, and TPS65 were upregulated. The expressions of monoterpenol glycosyltransferase (GT) 12, GT13, GT15, and GT20 were significantly enhanced in RDI-1S berries, whereas those of GT12, GT13, GT19, and GT20 were downregulated in RDI-2S berries. **Figure 4** reports the Pearson’s correlation coefficient between free/glycosylated monoterpenes and the relative expression of the TPSs involved in their biosynthesis. In particular, TPS54 and TPS59 were significantly and positively correlated with most glycosylated monoterpenes and negatively correlated with the free ones, whereas the opposite was observed for TPS35 (**Figure 4**; **Supplementary Tables S1**, **S2**). Despite few significant correlations, similar patterns were also observed for TPS31, TPS55, and TPS65. The main putative genes proposed for regulating the terpenes pathways are reported in **Figure 5**. The RsGT1 and RsGT-like1 regulatory genes were significantly correlated with TPS35, TPS56, and TPS65, whereas NAC71 and ABCG7 were significantly correlated with TPS34, TPS54, and TPS55. A positive correlation was also observed between MYB24 and TPS35 and between HMBPP and TPS34 and TPS56 (**Figure 5**; **Supplementary Tables S1**, **S2**). On the contrary, HY5, EPHX2, and AAP were significantly inversely correlated with TPS54, TPS55, and TPS59 (**Figure 5**; **Supplementary Tables S1**, **S2**).

**Figure 6:**
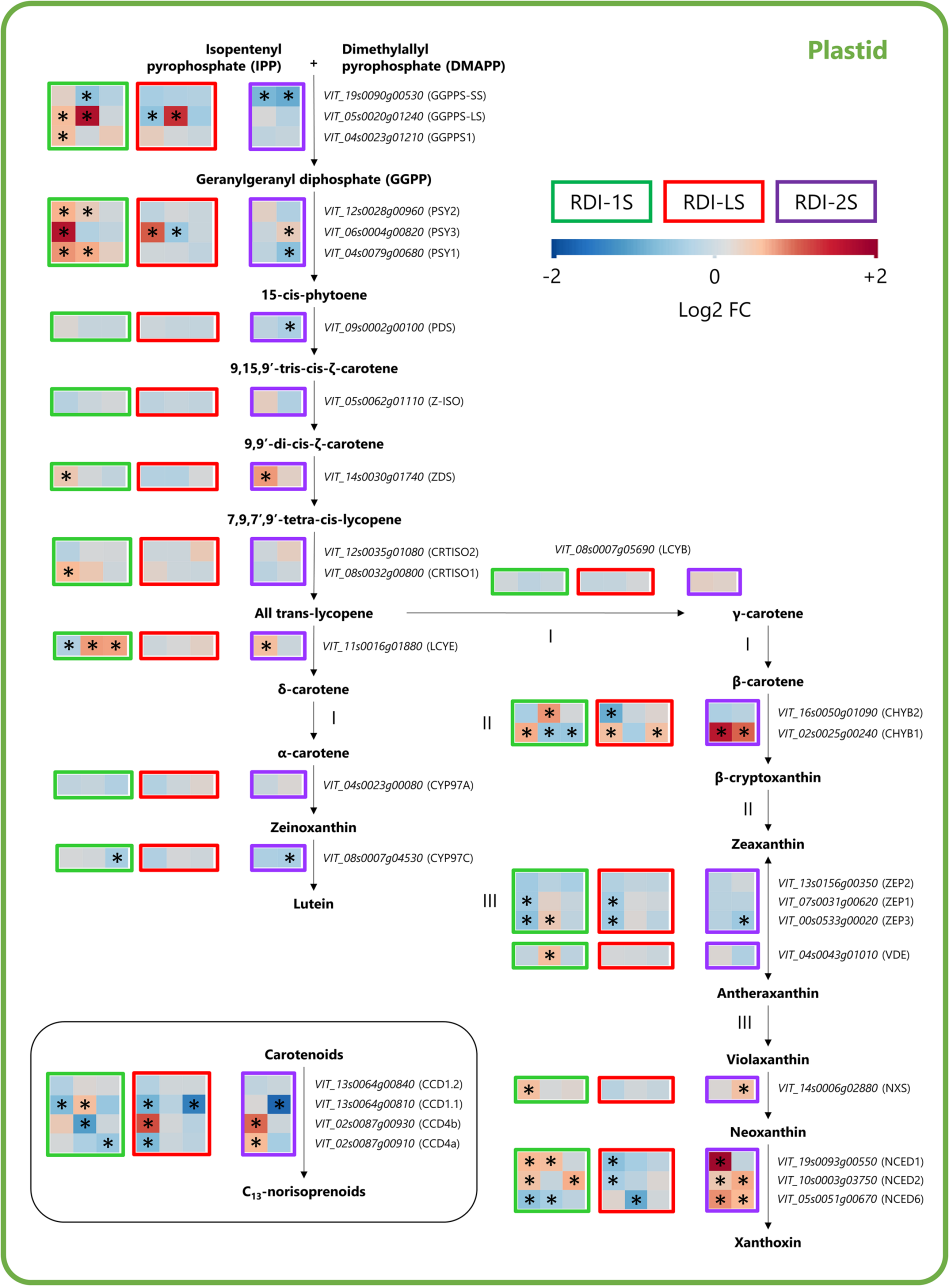
Modulation of the carotenoid pathway in berries of Sangiovese grapevines (*Vitis vinifera* L.) subjected to different irrigation regimes: RDI-1S, severe water deficit applied from berry pea size to beginning of veraison (green block); RDI-LS, water deficit applied during lag phase (red block); RDI-2S, severe water deficit applied from beginning of veraison to harvest (purple block). Log2FC (RDI/FI) levels of differential gene expression are presented at the beginning of veraison (left box), mid-ripening (central box), and harvest (right box). Asterisks identify significant differences (p < 0.05) between treatments. Roman numbers indicate commonly regulated steps of the pathway.

Throughout the carotenoid pathway, GGPPS-LS, GGPPS1, phytoene synthase genes (PSY1, PSY2, and PSY3), ζ-carotene desaturase (ZDS), carotenoid isomerases CRTISO1, and lycopene ε-cyclase (LCYE) were significantly overexpressed by RDI-1S especially at veraison and mid-ripening (**Figure 6**). Downstream the pathway, the RDI-1S also promoted the expression of violaxanthin de-epoxidase (VDE), neoxanthin synthase (NXS), and two nine-cis epoxycarotenoid dioxygenase (NCED1 and NCED2). The severe post-veraison water deficit enhanced the expression of ZDS and LCYE genes and β-carotene hydroxylase 1 (CHYB1), NXS, and all the NCEDs genes (**Figure 6**). The effect of RDI-LS on the carotenoid biosynthesis was less evident: GGPPS-LS and PSY3 were upregulated at mid-ripening and veraison, respectively, whereas zeaxanthin epoxidases (ZEP1 and ZEP3) and NCED1, NCED2, and NCED6 were downregulated (**Figure 6**). The carotenoid cleavage dioxygenase (CCD)1.1, CCD4a, and CCD4b, which are responsible for the C_13_-norisoprenoids biosynthesis, were differentially expressed in all the irrigation treatments (**Figure 6**).

## 4 Discussion

The results of the current study provide new evidence about the effect of the timing and intensity of water deficit on the concentration of berry glycosylated and free VOCs and the regulation of the relative biosynthetic pathways.

The phenological stage at which stress was imposed affected berry total VOCs (glycosylated+free) concentration. In both years, the highest berry total VOCs were measured in berries from vines subjected to water deficit from pea size through veraison, confirming previous results (Palai et al., 2022b). On the contrary, the post-veraison water deficit did not significantly affect the concentration of berry total VOCs, showing values similar to those measured in FI berries.

Despite the short period of water restriction, berries from RDI-LS vines had similar VOC concentrations than RDI-1 while maintaining unaltered berry size and titratable acidity with respect to control berries. Confirming our initial hypothesis, the pre-veraison period, and particularly the lag phase, was crucial for the regulation of VOC biosynthesis, as the water stress applied was able to modulate the relative pathways even after relief of stress (**Figures 3**, **6**). Previous findings indicated that the lag phase was a key stage in VOC biosynthesis regulation (Kalua and Boss, 2010; Matarese et al., 2013), but to the best of our knowledge, our results provide the first evidence on the effect of water deficit during the lag phase on berry VOCs. We can also speculate that the VOCs increment observed in RDI-1 berries was potentially induced by the days of water stress during the lag phase rather than the effect of the whole deficit period from berry pea size to veraison, although further work is necessary to test this hypothesis. We also cannot exclude a positive effect of the post-veraison re-watering applied in RDI-1 and RDI-LS vines. Indeed, the detrimental effect of prolonged water-deficit treatments on berry VOCs with respect to regimes that restored vine water status by supplying higher irrigation volumes has been reported in Muscat of Alexandria, Bobal, and Cabernet Sauvignon (Buesa et al., 2021; Lizama et al., 2021; Torres et al., 2022). Thus, the VOC increment measured in RDI-1 and RDI-LS berries could have been driven by high photosynthetic activity following relief of stress, which resumed carbon supply during berry ripening when VOCs were mainly synthesized (D’Onofrio, 2013; Godshaw et al., 2019; Yue et al., 2020).

The period when water stress was imposed differently affected the glycosylated and free forms of berry VOCs. Water deficit applied before veraison and during the lag phase induced the highest concentration of berry glycosylated VOCs, which quantitatively contributed the most to Sangiovese aroma profile (approximately 70% of berry total VOCs) as also observed in other varieties (Yue et al., 2020). The highest level of glycosylated VOCs measured in RDI-1S berries is consistent with the upregulation of many GTs of the monoterpenes MEP pathway, which were overexpressed during ripening when the irrigation was restored (**Figure 3**). On the other hand, the free VOC concentrations were higher in berries from vines subjected to water deficit during the lag phase and after veraison, even if this pattern was less clear and often not significant for individual free compounds. Considering that free volatile emissions play an active role under abiotic stress conditions as oxidative signaling and regulating the stress-induced senescence (Brilli et al., 2019; Boncan et al., 2020), the higher free VOCs of RDI-2 berries, which were harvested from vines still under water deficit, could be another response whereby vines cope with lack of water.

Free and glycosylated compounds were also differently affected by the growing season, as confirmed by PCA. Considering the free VOCs dataset, the component 2 of PCA discriminates samples between the 2 years (**Figure 2B**), whereas it does not consider glycosylated VOCs (**Figure 2A**). Thus, this finding allows to assume that while the glycosylated fraction was mainly determined by the irrigation regime, berry free VOCs were affected by both the growing season and vine water status.

The effect of water deficit on berry aroma compounds was also determined by the severity of the stress imposed. Significant differences on the concentration of many glycosylated compounds were measured between the severe and the moderate water deficit when the stress was applied before veraison (**Figure 1**). The significant positive linear correlation that was found confirms the hypothesis that glycosylated aroma compounds increased as pre-veraison water stress increased. This pattern of correlation is consistent with what was previously reported for cv. Sangiovese (Palai et al., 2022b). On the other hand, no significant differences were induced by severe or moderate stress after veraison, validating the relevance of the pre-veraison in VOCs biosynthesis modulation and indicating how the period of water shortage prevails on its severity. It is also worth noting that the free fraction was just slightly affected by water stress severity, suggesting a more complex modulation of free VOCs that included other factors (e.g., climatic conditions) besides irrigation as discussed above.

The effect of the timing and intensity of water stress on total free and glycosylated VOCs can also be extended to many key VOCs and on their biosynthetic pathways. In RDI-1S and RDI-LS grapevines, berry monoterpenes biosynthesis was enhanced by water deficit, particularly linalool, geraniol, α-terpineol, and their derivatives through the MVA and MEP pathways. Savoi et al. (2016) reported that pre-veraison water deficit increased the accumulation of free nerol, linalool, hotrienol, and α-terpineol in Tocai Friulano, whereas Wang et al. (2019) observed a higher concentration of free α-terpineol and linalool in Viognier berries from early (pre-veraison) water-stressed vines. The TPSs and GTs downstream terpene pathways were the most regulated genes, similar to results obtained in other varieties (Savoi et al., 2016; Yue et al., 2020). Moreover, especially in RDI-1S berries, many genes of the terpenes pathways were significantly overexpressed until the harvest date, which was far after water deficit was released (**Figure 3**). The linalool nerolidol synthase TPS54 was upregulated in RDI-1S berries in agreement with what was reported in Viognier and Tocai Friulano (Savoi et al., 2016; Wang et al., 2019), and it was upregulated during the last phase of ripening, consistent to its expression peak, as also observed in Moscato Bianco by Matarese et al. (2013). The higher linalool derivatives accumulated in RDI-1S berries was also induced by the overexpression of the linalool nerolidol TPS55 and of the (3*S*)-linalool synthase TPS56, which was upregulated in the later stage of ripening according to previous studies (Lin et al., 2019; Cramer et al., 2020). As observed by Liu et al. (2022) in table grape varieties, we did not detect TPS52, characterized as the only geraniol synthase in grape (Martin et al., 2010). This could be consistent to the developmentally specific transcripts level of TPS52, which peaked its expression in green berries (Wen et al., 2015), but decreased dramatically during ripening (D’Onofrio et al., 2018). The Pearson’s correlation analysis between differentially expressed genes and monoterpenes showed that TPS54, TPS55, and TPS59 were significantly positively and negatively related with the concentration of glycosylated and free monoterpenes, respectively, whereas the opposite was observed for TPS35. In particular, the TPS55 was significantly correlated with glycosylated linalool, whereas TPS59, recently suggested as involved in linalool biosynthesis (Liu et al., 2022), was also related to glycosylated geraniol, α-terpineol, and their derivatives. Despite to a lesser extent, similar correlations were observed for TPS54, proposed as linalool nerolidol synthase by Martin et al. (2010). The key role of TPS35 in linalool accumulation (Wang et al., 2021) was consistent to what we observed for linalool pyranoid oxides, even if only in the free form. The pairwise correlations between the expression of TPSs and many TFs are consistent to a possible role of the latter to regulate the TPS activity through free or glycosylated compounds. The drought-tolerant NAC71 transcription factor gene, previously associated with bound linalool derivatives (Costantini et al., 2017), was significantly and positively correlated with TPS54 and TPS55, which, in turn, were correlated to glycosylated monoterpenes (**Figure 4**). On the contrary, the MYBTKl1 protein, reported as highly correlated with free linalool (Costantini et al., 2017), was negatively correlated with TPS54 and TPS59. MYB24, proposed as a regulatory candidate for monoterpene pathways under drought and abiotic stress (Wong et al., 2016; Zhu et al., 2022), was positively correlated with TPS35, which in turn was correlated to free terpenes in agreement to Savoi et al. (2016). Overall, these findings suggest that some TPSs may play a role in the biosynthesis of free rather than glycosylated compounds. However, since we determined VOCs only at harvest for all the irrigation treatments, we cannot also exclude that these different modulations occurred due to a possible different sensitivity of single TPSs during berry development. Among GTs, GT7, GT14, and GT15 were functionally characterized as involved in the glycosylation of geraniol, nerol, citronellol, and linalool (Bönisch et al., 2014; Li et al., 2017). Among the other possible GTs involved, the putative GT20 seems to be consistent with the analytical data helping to explain the higher concentration of glycosylated monoterpenes (especially linalool derivatives) measured in RDI-LS berries with respect to the RDI-2S ones.

Pre-veraison and lag-phase water deficit increased C_13_-norisoprenoids. Although the carotenoid pathway revealed many regulatory steps mainly attributed to CCDs, NCED and PSY genes in water-stressed vines (Deluc et al., 2009; Savoi et al., 2016), the expression pattern of these genes between treatments does not seem to explain the different concentrations of C_13_-norisoprenoids measured at harvest. Indeed, many overexpressed genes throughout the pathway were observed also in RDI-2S berries, which, at harvest, had similar C_13_-norisoprenoid concentrations than FI ones. Therefore, we hypothesize that: i) the overexpression of carotenoids genes induced in RDI-2S berries during the water-deficit period was shifted to support the ABA biosynthesis (He et al., 2021) or photoprotective mechanisms by the xanthophyll cycle pool (Palliotti et al., 2015) rather than C_13_-norisoprenoids biosynthesis; ii) RDI-1S and RDI-LS treatments may have induced a higher accumulation of berry carotenoids (precursors of C_13_-norisoprenoids) that were mainly synthesized before veraison (thus, undetected in our analysis) and then broken down later during ripening into C_13_-norisoprenoids (Baumes et al., 2002). Alternatively, carotenoids in plants under abiotic stress could be diverted towards different pathways such as those responsible for the biosynthesis of apocarotenoids and strigolactones (Sun et al., 2022).

Water deficit also increased the shikimate VOC derivatives, which play a fundamental role in the pathways of specialized metabolites involved in the plant response to abiotic stresses, such as plant hormones, cofactors, and defense compounds (Widhalm and Dudareva, 2015). Their concentrations were enhanced especially when water deficit was imposed before veraison, according to what was recently reported in Sangiovese (Palai et al., 2022b). In wines produced from Verdejo and Merlot grapevines subjected to rainfed condition and water-deficit irrigation (35% ET_c_), respectively, a higher concentration of 4-vinylguaiacol and guaiacol was measured (Qian et al., 2009; Vilanova et al., 2019). The higher concentration of volatile phenols also observed in RDI-1S and RDI-LS berries could be associated with an overexpression of the phenylpropanoids pathway (Lin et al., 2019) consistently to the relative gene expression data previously reported in Palai et al. (2022a).

## 5 Conclusions

Water deficit differently affected berry VOCs according to the period when it was imposed. Higher total VOC concentrations at harvest were measured in berries from water-stressed vines before veraison and during lag phase, whereas no changes were detected when water deficit was imposed after veraison. This result was even more evident considering the glycosylated fraction, particularly glycosylated monoterpenes and C_13_-norisoprenoids, whereas free VOCs, which accounted for approximately 30% of total VOCs, was higher in berries from lag phase and post-veraison stressed vines. The severity of water stress played a key role as well, especially on glycosylated VOCs, which showed a positive correlation with the intensity of the stress experienced before veraison. The berry VOCs increment induced by the brief period of water shortage during lag phase allows to hypothesize the central role played by this stage in berry aroma compounds biosynthesis modulation, without the detrimental effect of pre-veraison water deficit on berry fresh weight and on titratable acidity. The gene expression analysis showed a wide upregulation of many TPSs and GTs of the terpenes MEP and MVA pathways, especially borne by berries from pre-veraison water stressed vines and even far after the water stress was released. Overall, these results contribute to the understanding of the effect of the timing and intensity of water deficit on berry VOCs accumulation, in order to manage irrigation to achieve high quality productions while optimizing water resources.

## Data availability statement

The data presented in the study are deposited in the NCBI sequence read archive (https://www.ncbi.nlm.nih.gov/sra), accession number PRJNA886074.

## Author contributions

GP participated in the design of the study, carried out the field data acquisition, carried out the laboratory analyses and part of the transcriptome data analysis, interpreted the results, and elaborated the manuscript draft and the final version. CD participated in the design of the study, provided funding and equipment, coordinated the laboratory analyses, and elaborated the manuscript draft and the final version. RG participated in the design of the study, provided funding and equipment, and critically reviewed the manuscript. GC participated in the design of the study, coordinated the field experiments, carried out the field data acquisition, and elaborated the manuscript draft and the final version. All authors contributed to the article and approved the submitted version.

## Supplementary material

Supplementary Figure 1 Total soluble solids (TSS) titratable acidity (TA), pH and berry fresh weight (FW) from veraison to harvest measured in 2019 **(A, C, E, G)** and 2020 **(B, D, F, H)** in Sangiovese grapevines (*Vitis vinifera* L.) subjected to six different irrigation protocols (FI, full irrigated from pea-size berry through harvest; RDI-1S and RDI-1M, severe and moderate water deficit applied from pea-size berry through veraison; RDI-LS, water deficit applied during lag-phase; RDI-2S and RDI-2M, severe and moderate water deficit applied from veraison through harvest). Values are means ± standard deviations of three replicates per treatment (n = 3). The grey box indicates the harvest period.

Supplementary Figure 2 Skin to pulp ratio and specific skin weight measured in 2019 **(A, B)** and 2020 **(C, D)** in Sangiovese (*Vitis vinifera* L.) grapevines subjected to six different irrigation regimes (FI, full irrigation from berry pea-size to harvest; RDI-1S and RDI-1M, severe and moderate water deficit applied from berry pea-size to beginning of veraison; RDI-LS, water deficit applied during lag-phase; RDI-2S and RDI-2M, severe and moderate water deficit applied from beginning of veraison to harvest). Values are means ± standard deviation of three replicates. Different letters indicate honest significant differences (HSD) between irrigation treatments after analysis of variance (ANOVA) within each date (P < 0.05).

Supplementary Table 1 Correlation coefficients between free monoterpenes, glycosylated monoterpenes, monoterpenes regulators genes and the relative expression of monoterpenes synthases within all irrigation treatments in 2020 year.

Supplementary Table 2 P-values of correlation probability between free monoterpenes, glycosylated monoterpenes, monoterpenes regulators genes and the relative expression of monoterpenes synthases within all irrigation treatments in 2020 year.
