# Supplementary figures and images for: Water deficit before veraison is crucial in regulating berry VOCs concentration in Sangiovese grapevines
Source: Front Plant Sci. 2023 Feb 20;14:1117572. doi: 10.3389/fpls.2023.1117572 (PMC9986437; doi:10.3389/fpls.2023.1117572)

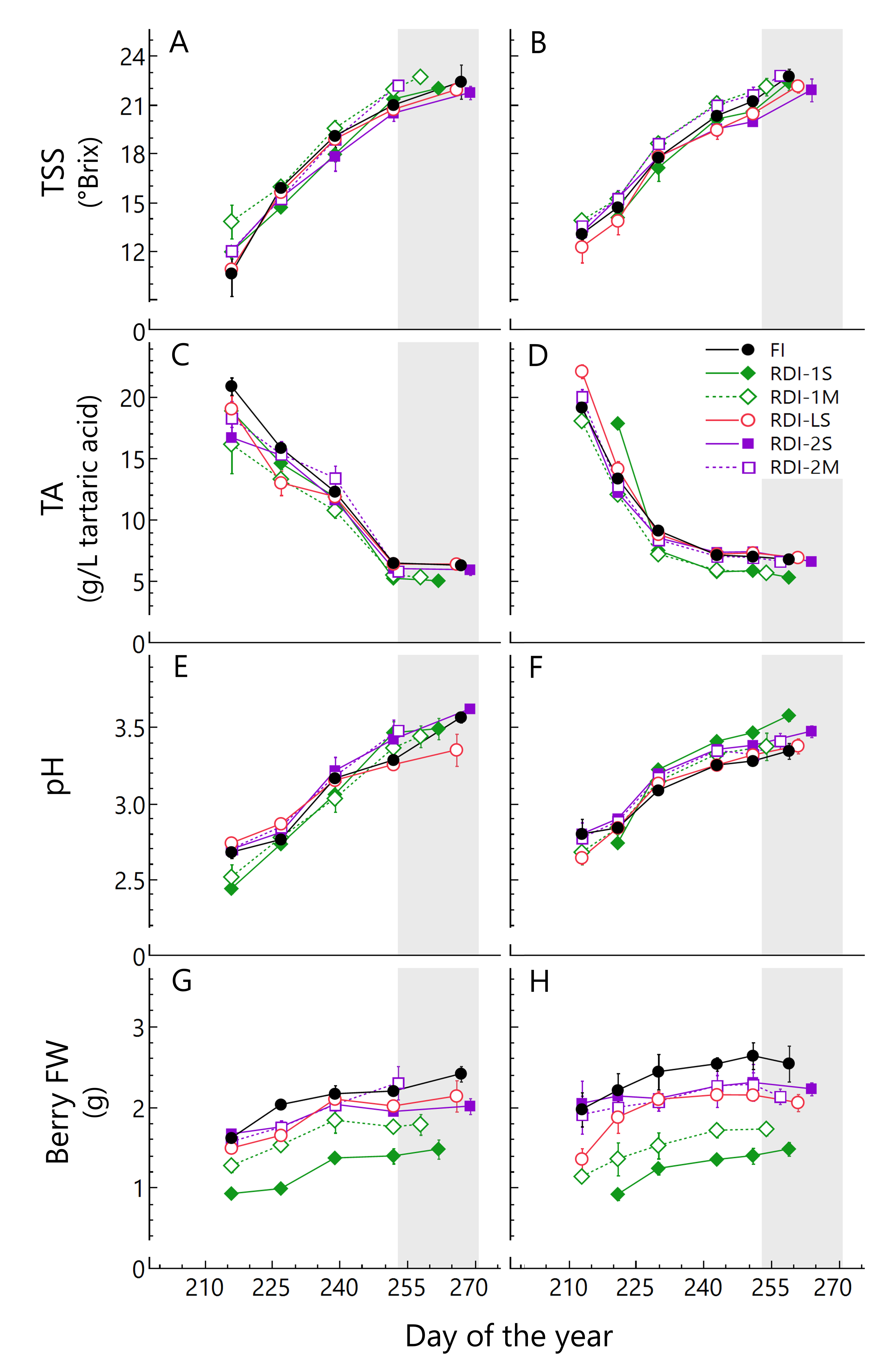

Supplement: Supplementary Figure 1 — Total soluble solids (TSS) titratable acidity (TA), pH and berry fresh weight (FW) from veraison to harvest measured in 2019 (A, C, E, G) and 2020 (B, D, F, H) in Sangiovese grapevines (Vitis vinifera L.) subjected to six different irrigation protocols (FI, full irrigated from pea-size berry through harvest; RDI-1S and RDI-1M, severe and moderate water deficit applied from pea-size berry through veraison; RDI-LS, water deficit applied during lag-phase; RDI-2S and RDI-2M, severe and moderate water deficit applied from veraison through harvest). Values are means ± standard deviations of three replicates per treatment (n = 3). The grey box indicates the harvest period. [file Image_1.tif]

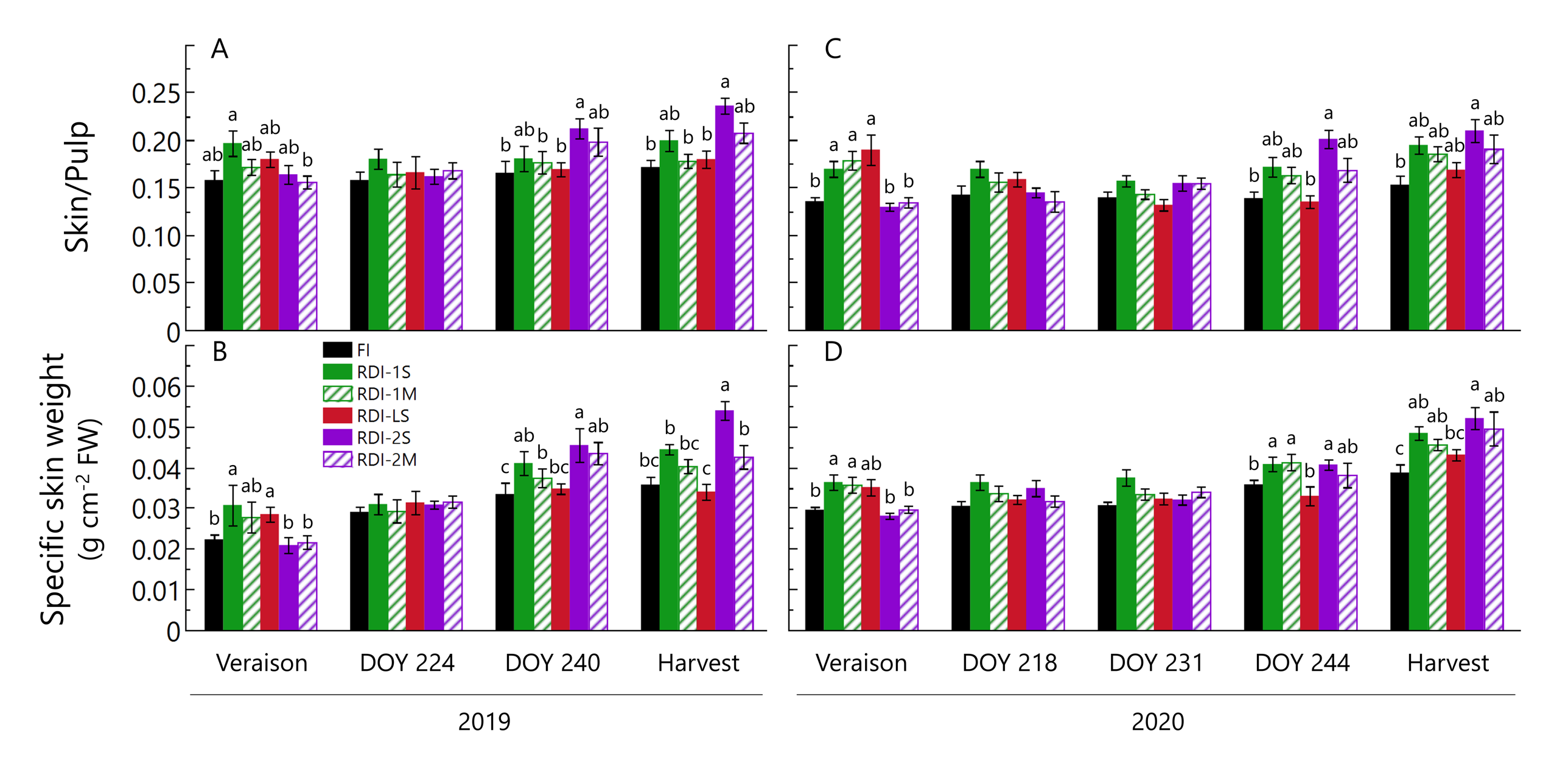

Supplement: Supplementary Figure 2 — Skin to pulp ratio and specific skin weight measured in 2019 (A, B) and 2020 (C, D) in Sangiovese (Vitis vinifera L.) grapevines subjected to six different irrigation regimes (FI, full irrigation from berry pea-size to harvest; RDI-1S and RDI-1M, severe and moderate water deficit applied from berry pea-size to beginning of veraison; RDI-LS, water deficit applied during lag-phase; RDI-2S and RDI-2M, severe and moderate water deficit applied from beginning of veraison to harvest). Values are means ± standard deviation of three replicates. Different letters indicate honest significant differences (HSD) between irrigation treatments after analysis of variance (ANOVA) within each date (P < 0.05). [file Image_2.tif]
